# Spectrum of Neuroimaging Findings in CNS Lymphoproliferative Disorders

**DOI:** 10.3390/diagnostics16182932

**Published:** 2026-09-10

**Authors:** Matheus Hideki Taborda, João Rudolfo Kleinubing, Diego Ricardo Lodi Lauriano, João Victor de Oliveira Souza, Leonardo Kami, James Henrique Yared, Bruno Augusto Telles, Erasmo Barros da Silva, Marcela Santos Cavalcanti, Ricardo Ramina, Bernardo Corrêa de Almeida Teixeira

**Affiliations:** 1 Health Sciences and Internal Medicine Postgraduate Program, Health Sciences Deparment, Federal University of Paraná (PPGMICS-UFPR), Rua General Carneiro 181, Curitiba 82860-140, Paraná, Brazil; matheushideki@hotmail.com; 2 Department of Neuroradiology, Neurological Institute of Curitiba, Rua Jeremias Maciel Perretto, 300, Curitiba 81210-310, Paraná, Brazildiego.lodi@hotmail.com (D.R.L.L.); jvictoroliveirasouza@gmail.com (J.V.d.O.S.); leolkt28@gmail.com (L.K.); jamesyared@hotmail.com (J.H.Y.); brunoaugustotelles@hotmail.com (B.A.T.); 3 Department of Neurosurgery, Neurological Institute of Curitiba, Rua Jeremias Maciel Perretto, 300, Curitiba 81210-310, Paraná, Brazilramina@hospitalinc.com.br (R.R.); 4 Department of Pathology, Neurological Institute of Curitiba, Rua Jeremias Maciel Perretto, 300, Curitiba 81210-310, Paraná, Brazil; marcellascava@gmail.com

**Keywords:** neuroimaging, magnetic resonance imaging, CNS lymphoproliferative disorders, CNS lymphoma, large B-cell lymphoma

## Abstract

Central nervous system (CNS) lymphoproliferative disorders comprise a heterogeneous group of rare entities that present major diagnostic and therapeutic challenges. Primary CNS large B-cell lymphoma (LBCL) is the most common form and the second most frequent primary CNS tumor after gliomas. The 5th edition of the World Health Organization Classification of Hematolymphoid Tumours introduced updated terminology, including primary LBCL of immune-privileged sites and lymphomas arising in immunodeficiency/dysregulation, along with less common variants such as intravascular large B-cell lymphoma, lymphomatoid granulomatosis, lymphomatosis cerebri, secondary CNS lymphoma, mucosa-associated lymphoid tissue (MALT) lymphoma of the dura, and T-cell/NK-cell lymphomas. Immune status, Epstein–Barr virus association, and imaging features may assist differentiation, although biopsy remains essential for definitive diagnosis. On MRI, lesions are typically periventricular or involve the basal ganglia, showing low T2 signal intensity, restricted diffusion, and intense enhancement. Spectroscopy demonstrates elevated choline/N-acetylaspartate ratios with lipids, and perfusion is generally lower than in other neoplasms. Corticosteroid administration before biopsy should be avoided due to potential cytolysis of tumor cells. Major differential diagnoses include glioblastoma, metastases, demyelinating lesions, and infections such as toxoplasmosis, fungal abscess, and tuberculosis. Treatment may cause neurotoxicity and immunosuppression, predisposing patients to opportunistic infections.

## 1. Introduction

Central nervous system lymphoma (CNSL) is a rare but aggressive malignancy that presents major diagnostic and therapeutic challenges in neuro-oncology. Lymphomas are the second most common primary CNS tumors after gliomas [1], and timely recognition is essential, as management differs substantially from other CNS neoplasms. Both primary and secondary CNSL are most often of B-cell origin, with diffuse large B-cell lymphoma (DLBCL) being the predominant subtype [2], though several additional histologic variants exist with distinct clinical and imaging characteristics.

**Figure 1:**
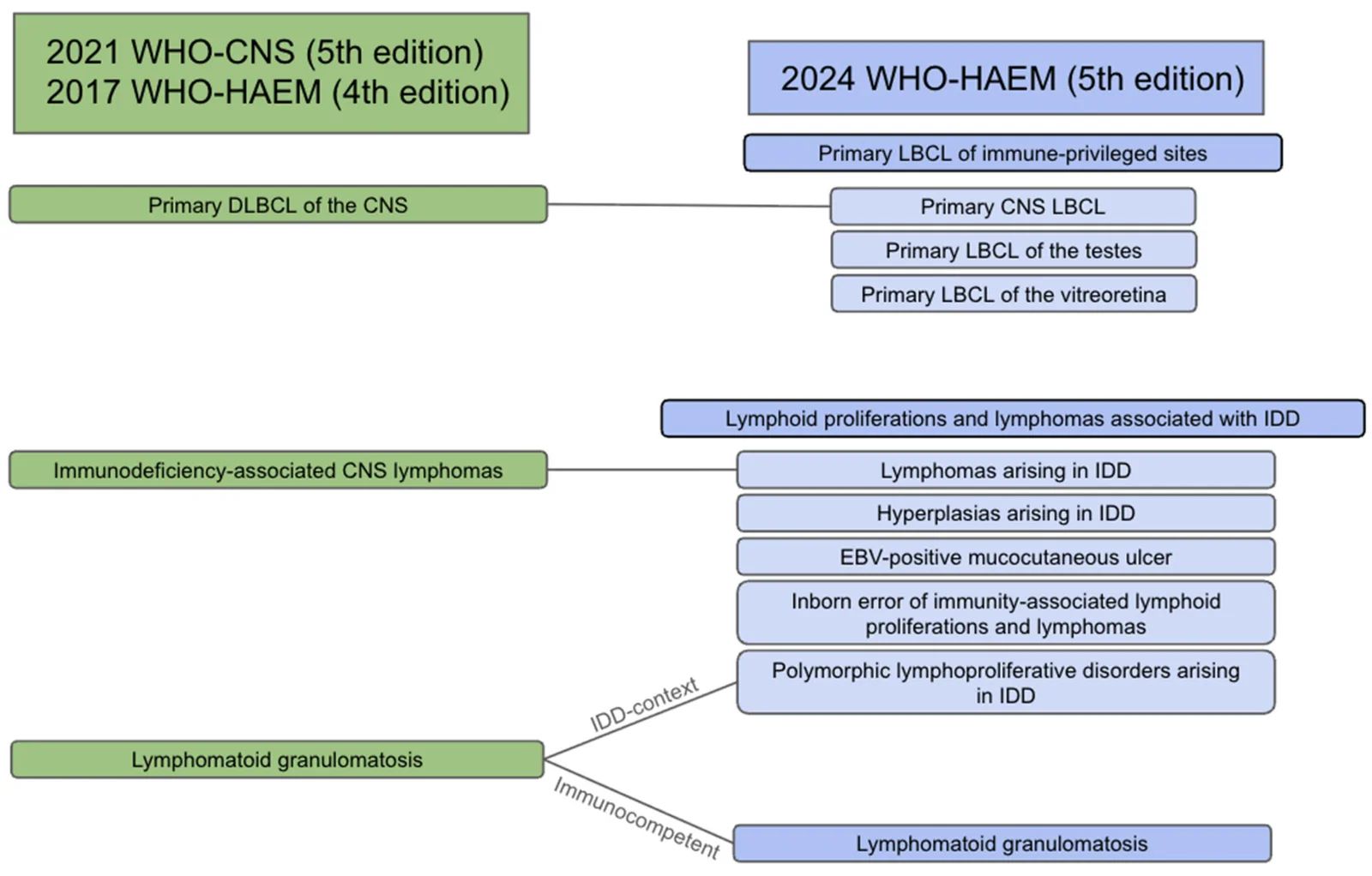
Main changes regarding CNS lymphoproliferative disorders’ nomenclature in the 2024 WHO-HAEM classification. Note: WHO-CNS = World Health Organization Classification of Tumors of the Central Nervous System, WHO-HAEM = World Health Organization Classification of Haematolymphoid Tumours, IDD = immune deficiency/dysregulation, DLBCL = diffuse large B-cell lymphoma, LBCL = large B-cell lymphoma.

Lymphomas are classified by cell of origin. The 5th edition of the World Health Organization (WHO) Classification of Tumours of the Central Nervous System (WHO-CNS) includes both common and rare subtypes [2], while the Classification of Haematolymphoid Tumours (WHO-HAEM5) [3] provides a more detailed categorization (Figure 1). Unlike the 4th edition, which emphasized histologic and clinical criteria, WHO-HAEM5 integrates molecular and cytogenetic data, aligning hematolymphoid classification with updates across other organ systems [3].

Lymphoid neoplasms are categorized using an integrated framework that combines clinical information, imaging data, and diverse laboratory findings, with microscopic morphology supported by immunophenotypic analysis forming the foundation of diagnostic decision-making. In recent decades, advances in genetic and genomic testing have profoundly reshaped this system, enabling the identification of new lymphoma entities and more accurate subtyping based on their biological origins [3].

Primary DLBCL of the CNS is now classified as primary CNS large B-cell lymphoma (PCNS-LBCL) and is part of the primary large B-cell lymphoma of immune-privileged sites (IP-LBCL) category in WHO-HAEM5, together with lymphomas of the vitreoretina and testes in immunocompetent individuals. This change reflects shared biologic and molecular features among these entities. IP-LBCL arises within immune-privileged sites protected by specialized barriers such as the blood–brain barrier, although similar lymphomas have been reported in the breast and skin [3].

Another key update concerns lymphoid proliferations and lymphomas associated with immune deficiency or dysregulation (IDD-lymphomas). The new nomenclature uses a three-part designation: histologic type (e.g., LBCL or polymorphic lymphoproliferative disorder), viral association, and immune deficiency context (e.g., HIV, post-transplant, or immune senescence). IDD-lymphomas now encompass tumors arising in diverse immunodeficiency states, including post-transplant, iatrogenic, and primary immune disorders. Epstein–Barr virus (EBV) status plays a critical role in their classification [3] (Table 1).

IP-LBCL and IDD-lymphomas together constitute most CNSL cases. Both are morphologically classified as LBCLs composed of large, atypical, monomorphic cells expressing mature B-cell markers. However, IDD-lymphomas may exhibit EBV-related protein expression, such as latent membrane protein 1 (LMP1) and EBV-encoded small RNAs (EBER) [2].

Lymphomatoid granulomatosis is another EBV-associated lymphoproliferative disorder but differs by its mixed cellular composition of T cells, plasma cells, and atypical B cells. Grading is determined by the number of atypical B cells. Intravascular large B-cell lymphoma (IVL) shares morphologic and immunohistochemical features with LBCL but is confined to the vascular lumina with minimal parenchymal invasion [2].

Rarer CNS lymphomas include mucosa-associated lymphoid tissue (MALT) lymphoma of the dura and other low-grade B-cell lymphomas, which are small-cell neoplasms with indolent behavior. T-cell lymphomas are exceedingly rare and encompass anaplastic large cell lymphoma and other T-cell/NK-cell variants [2].

The clinical presentation of CNSL varies by lesion location, mass effect, and peritumoral edema. Focal neurologic deficits such as motor or sensory impairment are common, while headache—often secondary to increased intracranial pressure—is the most frequent nonfocal symptom. Neuropsychiatric changes, cognitive decline, cranial neuropathies, and meningeal signs may occur with leptomeningeal spread [4]. Seizures are less frequent but may develop, particularly with cortical involvement, which is more typical of gliomas than lymphomas that favor deep structures.

Although biopsy remains the diagnostic gold standard, cerebrospinal fluid (CSF) analysis may reveal lymphocytic pleocytosis (34–60%), low glucose (10–54%), and elevated protein (>70%) [5]. Cytomorphologic examination is the reference method for detecting leptomeningeal dissemination but has limited sensitivity (2–32%) [5].

This narrative review provides a comprehensive overview of CNS lymphoproliferative disorders, emphasizing histopathologic subtypes and their imaging features based on the latest literature and current WHO classifications. While the 2021 WHO-CNS offers a simplified framework, understanding the molecular underpinnings of CNSL is essential for radiologists. The discussion covers IP-LBCL, IDD-lymphomas, intravascular large B-cell lymphoma, lymphomatoid granulomatosis, and lymphomatosis cerebri, followed by secondary CNS lymphoma, MALT lymphoma of the dura, and T-cell/NK-cell lymphomas. Finally, differential diagnoses, treatment strategies, and potential complications are reviewed. All materials and images are original and were retrospectively obtained from a tertiary neurosurgical referral center in Brazil.

## 2. Primary CNS Large B-Cell Lymphoma (PCNS-LBCL)

As of the new WHO-HAEM5 classification, PCNS-LBCL is now part of the primary B-cell lymphoma of immune-privileged sites (IP-LBCL), along with LBCL of the vitreoretina and LBCL of the testes [3]. The term primary CNS lymphoma is no longer recommended.

PCNS-LBCL is a subtype of non-Hodgkin lymphoma accounting for approximately 80–85% of CNSL. It is rare and aggressive, with an incidence of 0.5–1 case per 100,000 persons per year, and is confined to the CNS—including brain, spinal cord, and leptomeninges—without systemic involvement at diagnosis. The vitreoretina may be involved either primarily or as a site of relapse, and CNS involvement has also been reported following testicular LBCL [3]. The mean age at diagnosis is 63 years, with no sex predilection. Reported overall survival rates at 1, 3, and 5 years are 50%, 36%, and 27%, respectively, in patients treated with chemotherapy alone [6].

PCNS-LBCL typically arises in immunocompetent individuals and is typically Epstein–Barr virus (EBV)-negative. It exhibits a post-germinal-center B-cell immunophenotype, usually negative for CD10 and positive for MUM1 and BCL6 and concomitant MYD88 and CD79B mutations. Double expression of MYC and BCL2 in large B-cell lymphoma (DEL) is considered an independent high-risk prognostic factor. Although DEL is described in a specific section of the WHO-HAEM5, its presence in the context of IP-LBCL overrides the genetic classification [3].

### 2.1. Imaging Features

PCNS-LBCL usually presents as a single lesion or, in 30–35% of cases, as multiple parenchymal lesions with homogeneous enhancement. On CT, lesions appear hyperdense due to their high nuclear-to-cytoplasmic ratio and limited extracellular space. MRI typically demonstrates restricted diffusion on diffusion-weighted imaging (DWI) and low T2 signal intensity consistent with high cellularity. The T2 signal may be sufficiently low to reduce the hyperintensity seen on DWI, producing the *T2 blackout effect*—an important diagnostic pitfall. In such cases, ADC mapping is essential, as DWI may appear isointense [7].

**Figure 2:**
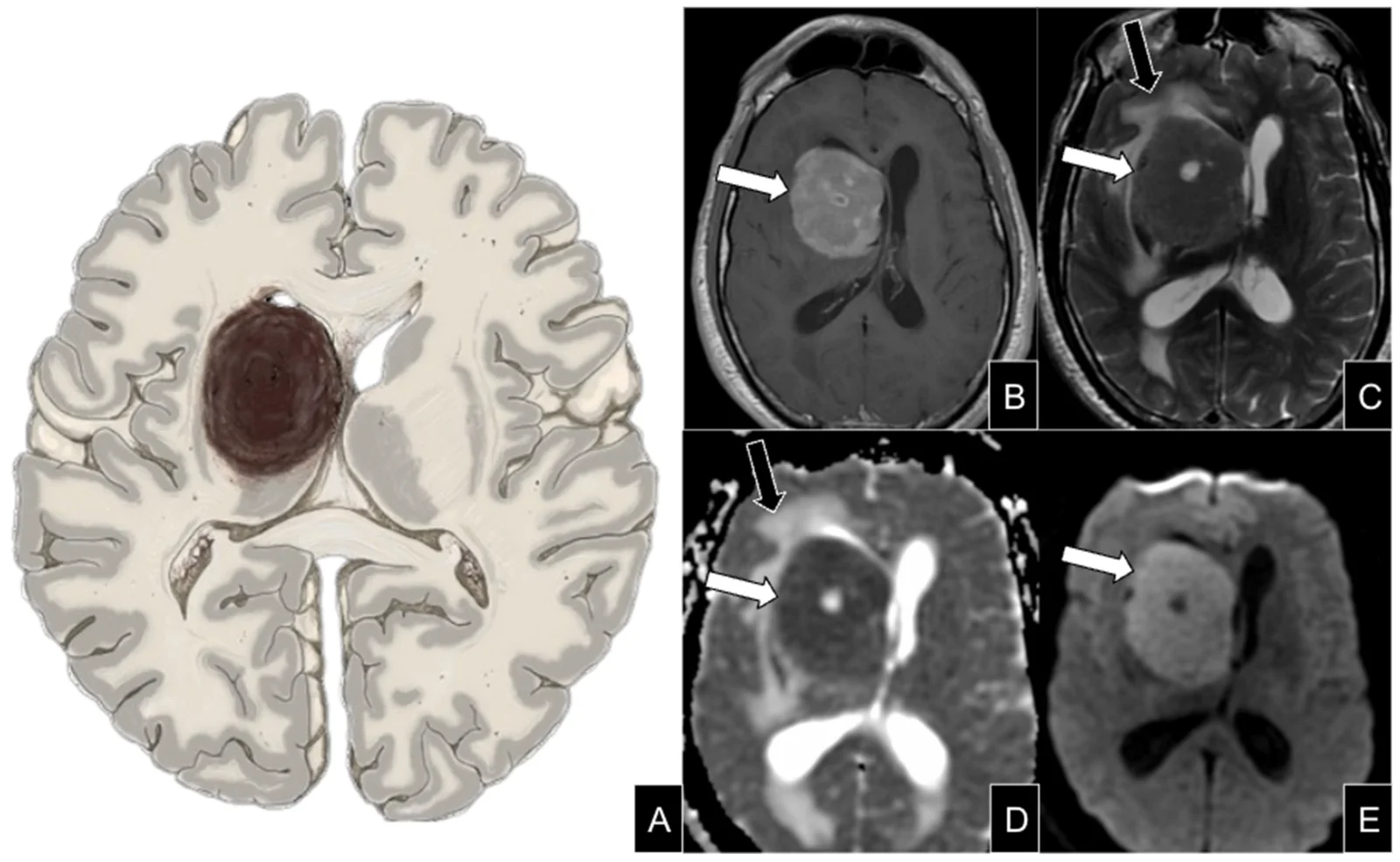
Large B-cell lymphoma of immune-privileged sites. Schematic representation (**A**) and axial contrast-enhanced T1-weighted (**B**), T2-weighted MRI (**C**), DWI (**D**), and ADC map (**E**) in an immunocompetent patient with a single supratentorial basal ganglia lesion (white arrows) with homogeneous contrast-enhancement, low T2 signal intensity, and restricted diffusion, reflecting the solid nature due to high cellularity. Also note peritumoral vasogenic edema (black arrows).

Perilesional edema is frequently observed, contributing to mass effect and associated symptoms. Susceptibility-weighted imaging (SWI) may show small hemosiderin deposits once thought to be absent in CNS lymphoma but now recognized as possible, though nonspecific [8]. CNS lymphoma is predominantly supratentorial (80%), with predilection for the basal ganglia, periventricular white matter, midline structures, and corpus callosum—key features distinguishing it from other CNS neoplasms [4] (Figure 2).

**Figure 3:**
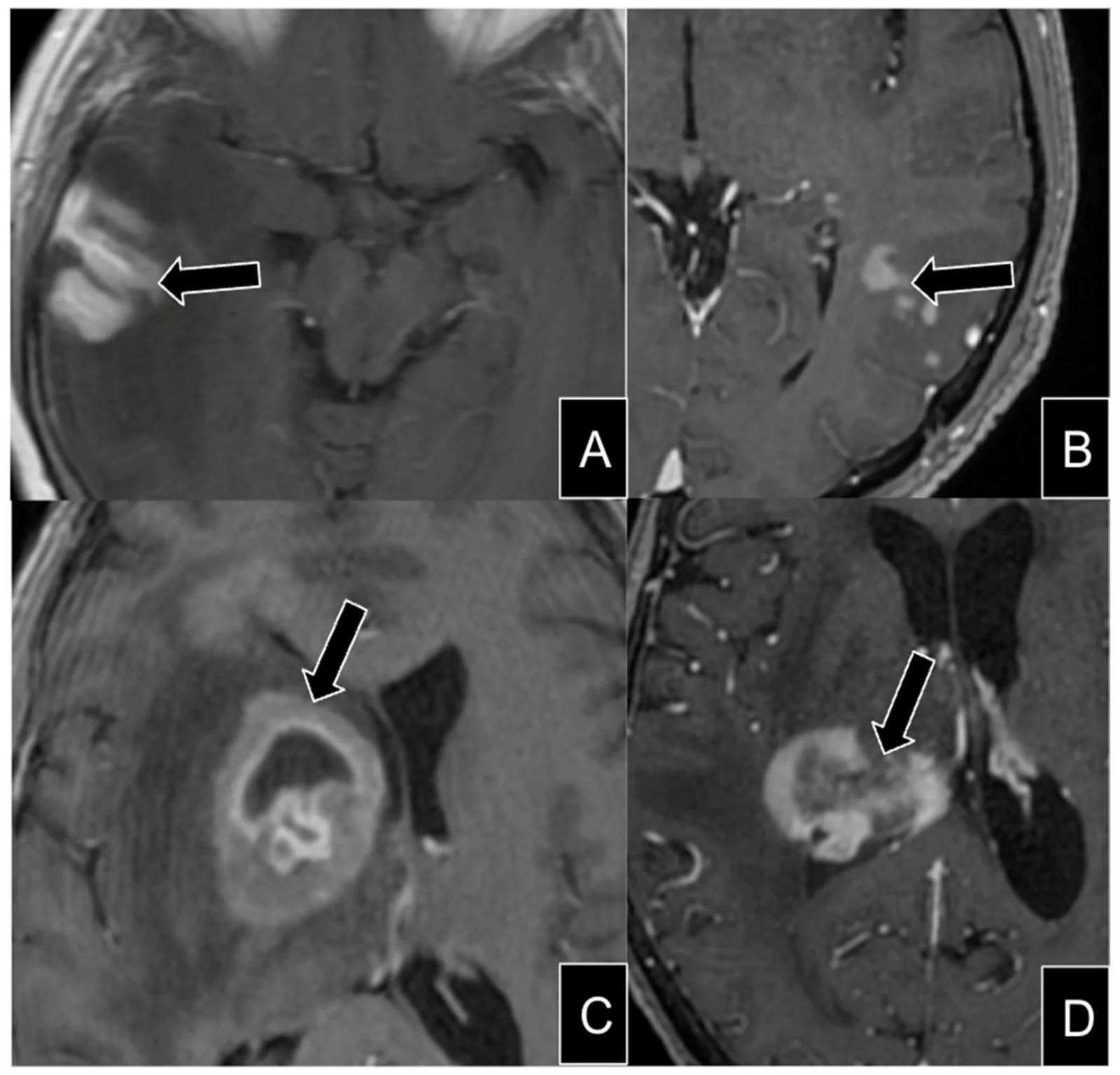
Enhancement pattern of large B-cell lymphoma of immune-privileged sites. Axial MRI showing variable enhancement patterns (black arrows), including gyriform (**A**), punctiform (**B**), nodular with cystic component (**C**), irregular and heterogeneous (**D**).

Although PCNS-LBCL classically demonstrates homogeneous enhancement, atypical patterns—gyriform, irregular, punctate, or heterogeneous with necrosis—may occur, the latter resembling glioblastoma [9]. Lesions with irregular enhancement or necrosis may mimic high-grade glioma or metastasis, delaying treatment (Figure 3). A recent study in immunocompetent patients reported heterogeneous and ring-like enhancement in 24.8% and 9.8% of cases, respectively, suggesting that necrotic variants are more common than previously thought [10].

**Figure 4:**
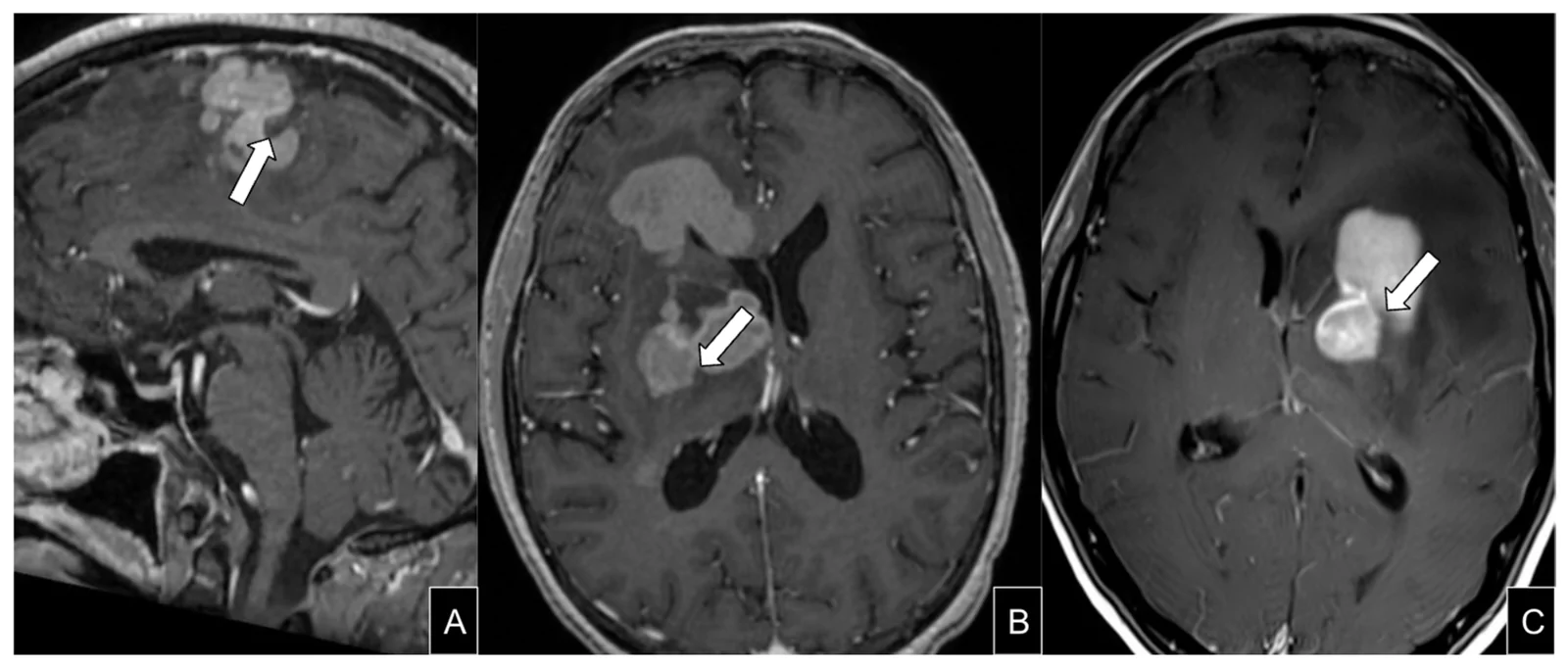
Notch sign. Sagittal (**A**) and axial (**B**,**C**) T1 contrast-enhanced MRI in patients with histological diagnosis of LBCL-IP. The “notch sign” is characterized by a depression along the outer margins of the lesion, which can be appreciated in multiple planes and usually better seen in post contrast sequences (white arrows).

The *notch sign*, a focal depression along the lesion margin, is another potential imaging feature, particularly in periventricular lesions (Figure 4). In small case series, it was considered a specific sign [11], more commonly observed in patients with PCNS-LBCL rather than secondary CNSL [12]. Its presence, especially when associated with restricted diffusion and low perfusion, should raise suspicion for PCNS-LBCL.

### 2.2. Diagnosis and Differential Diagnosis—Advanced and Functional Imaging

**Figure 5:**
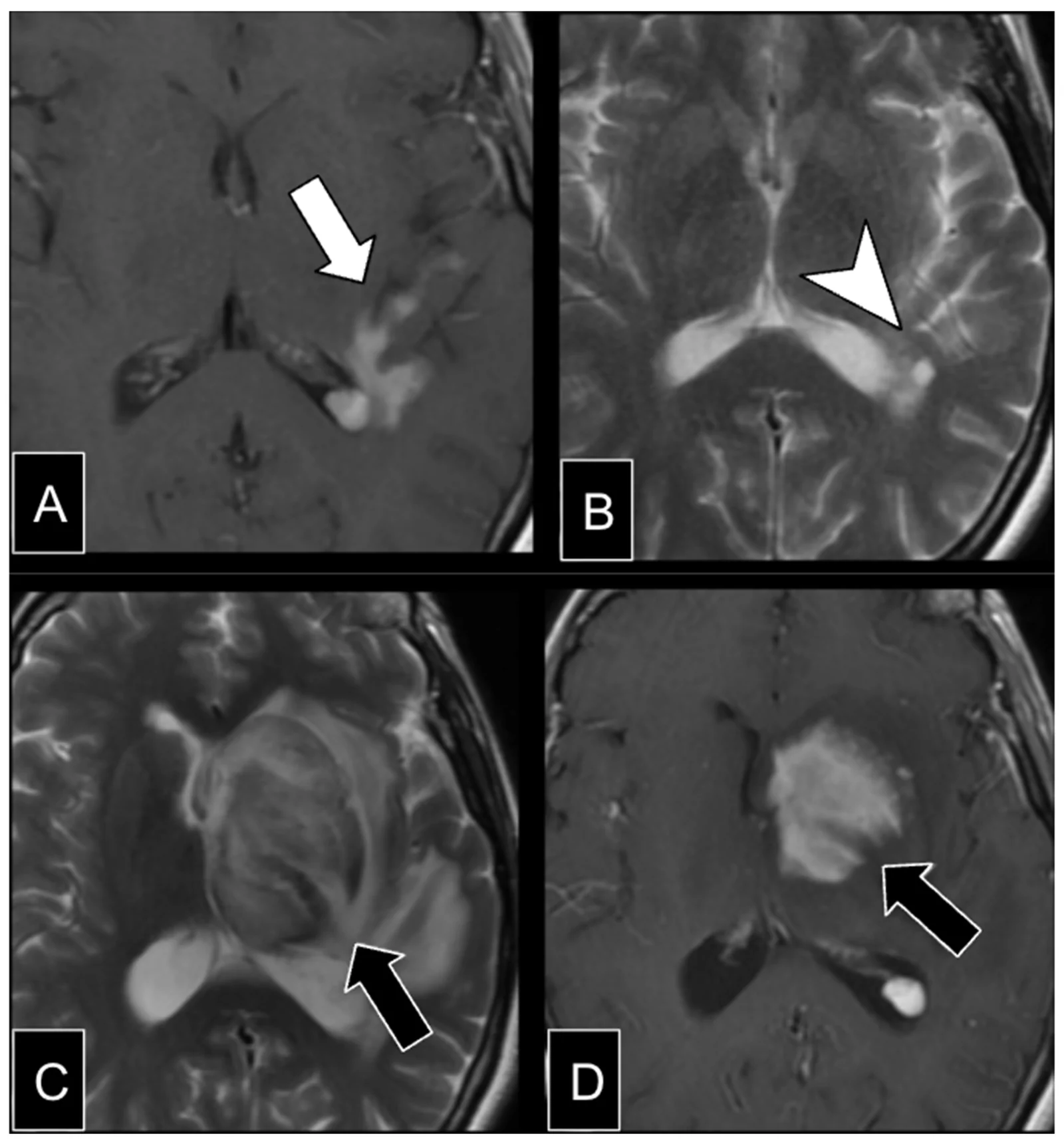
Impact of corticotherapy in CNSL diagnosis. (**A**) Axial contrast-enhanced T1-weighted imaging in a patient with dysarthria shows a periventricular irregular enhancing lesion (white arrow). (**B**) Axial T2-weighted MRI shows complete regression occurred after 3 months of corticotherapy. Stereotaxic biopsy (arrowhead) showed non-neoplastic inflammatory infiltrate. (**C**,**D**) Follow-up after 2 years shows appearance of a basal ganglia contrast-enhancing mass lesion with vasogenic edema, suggesting lymphoma. Also note the low T2 signal and the notch sign (black arrow).

Despite imaging advances, definitive diagnosis requires stereotactic biopsy. Corticosteroids should be avoided before biopsy, as they can obscure diagnostic findings by transiently reducing or eliminating neoplastic lymphoid cells through apoptosis and inflammation reduction, causing partial lesion regression (Figure 5) [13]. Differential diagnoses include gliomas, metastases, tumefactive demyelination, and infections such as neurotoxoplasmosis, fungal abscess, and tuberculosis.

Quantitative diffusion studies show that the mean ADC values of the solid contrast-enhancing regions in primary CNS lymphoma are significantly lower—typically 0.54–0.71 × 10^−3^ mm^2^/s—compared with 0.78–1.4 × 10^−3^ mm^2^/s in glioblastomas [14].

MR spectroscopy (MRS) typically demonstrates elevated choline (Cho) and reduced N-acetylaspartate (NAA), though these findings are nonspecific and overlap with other high-grade neoplasms. Markedly increased lipid resonances, reflected by a high lipid-to-creatine (Lip/Cr) ratio in solid tumors without macroscopic necrosis, are characteristic of CNS lymphoma and can help distinguish it from astrocytomas. Although glioblastomas with necrosis may show elevated lipids, the combination of high lipid levels and a markedly increased Cho/Cr ratio—usually exceeding those in astrocytomas—strongly favors CNSL [15]. Myo-inositol resonance, commonly observed in astrocytomas, aids in their distinction from lymphoma. MRS may also demonstrate abnormal spectra extending beyond the enhancing lesion, reflecting the infiltrative growth pattern of lymphoma [16].

Dynamic-susceptibility-contrast perfusion-weighted imaging (DSC-PWI) quantifies tumor microvasculature through parameters such as relative cerebral blood volume (rCBV) [17]. Gliomas and metastases typically show elevated rCBV, whereas CNS lymphoma demonstrates low rCBV due to minimal angiogenesis. This pattern is attributed to angiocentric growth, with tumor cells clustering around preexisting vessels, expanding perivascular spaces, and causing contrast leakage without neovascularization [18].

**Figure 6:**
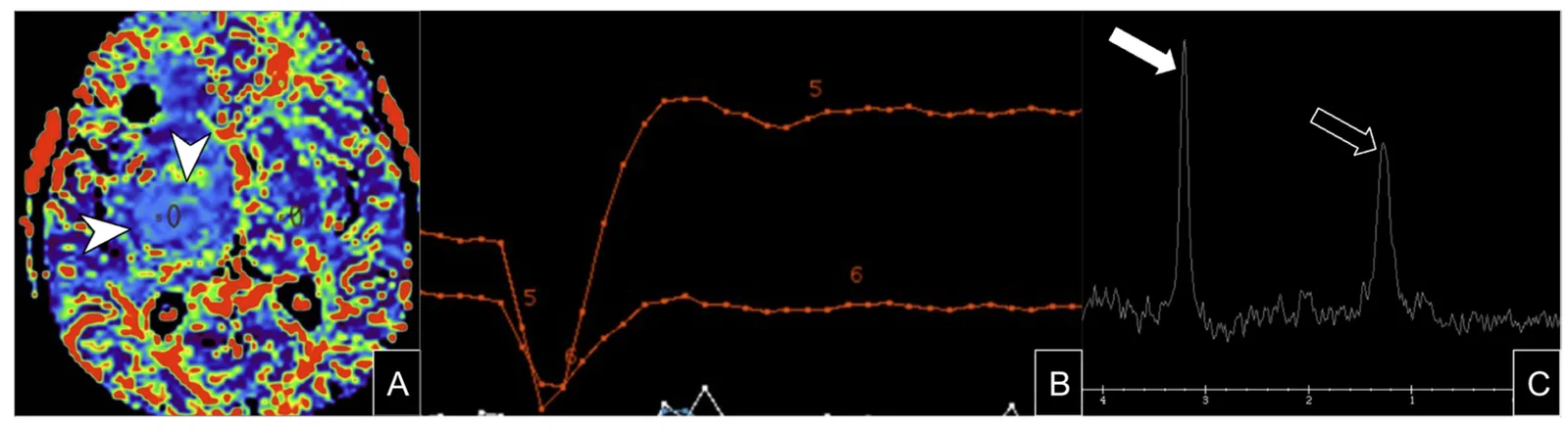
Dynamic-susceptibility-contrast perfusion-weighted imaging (DSC-PWI) in LBCL-IP. Axial DSC-PWI in a patient showing relatively lower rCBV (white arrowheads) compared to other neoplasms (**A**), with overshooting of the time–intensity curve above baseline (**B**), which is relatively specific to CNS lymphomas, although has limited sensitivity. MRS showing high choline (white arrow) with lipid peak (black arrow) at 144 ms TE (**C**). ROI and curve 5 are in the lesion and 6 is in the contralateral normal parenchyma.

A characteristic perfusion pattern in lymphoma is overshooting of the signal intensity recovery curve, where postcontrast signal exceeds the precontrast baseline after the first contrast pass. This phenomenon, quantified by the percentage signal recovery (PSR), is high in up to 79% of lymphomas and intermediate to low in gliomas and metastases, where perfusion curves typically remain below baseline (Figure 6) [19]. PSR shows less overlap among tumor types than rCBV and may be a more accurate discriminator [18].

Dynamic contrast-enhanced (DCE) MRI assesses blood–brain barrier permeability, most commonly using the transfer constant (Ktrans). PCNS-LBCL generally exhibits higher Ktrans values than glioblastoma or metastases. Lower pretreatment Ktrans values have been linked to faster disease progression. DCE-MRI can also monitor treatment response by quantifying vascular permeability changes, emphasizing its role as a noninvasive biomarker [17].

### 2.3. Prognostic Biomarkers

MRI-derived biomarkers have prognostic value. Low pretreatment ADC (<384 × 10^−6^ mm^2^/s) and rCBV (<1.43)—indicating high cellularity and hypoperfusion—are independently associated with poor outcomes. Combined, these parameters identify a subgroup with particularly poor prognosis. Additional adverse factors include infratentorial location, initial tumor volume > 11.4 cm^3^, and nonenhancing T2-FLAIR hyperintensities that may represent occult infiltration but often regress after therapy. These findings support incorporating metabolic and functional imaging into CNS lymphoma assessment and prognostic models [20].

## 3. Lymphomas Arising in Immunodeficiency/Dysregulation (IDD-Lymphoma)

IDD represents a newly defined group of diseases in the WHO-HAEM5, encompassing all lymphoid proliferations and lymphomas associated with immune deficiency and dysregulation that were previously classified elsewhere. It includes five distinct entities: hyperplasias, polymorphic lymphoproliferative disorders, EBV-associated mucocutaneous ulcer, IDD-lymphomas, and lymphomas/lymphoproliferative disorders linked to inborn errors of immunity. The IDD-lymphoma category now includes diseases arising in post-transplant and primary immunodeficiencies, as well as those previously described as CNSL associated with HIV infection in WHO-HAEM4 and WHO-SNC. Another major IDD context recently recognized is iatrogenic immunodeficiency, secondary to increasing use of multichemotherapy, immunotherapy, immunomodulatory agents, and next-generation immunosuppressants [3].

A key factor in the pathogenesis of these lymphomas is EBV, which plays a central role in tumor development. EBV or human herpesvirus 8 (HHV-8) positivity is observed in most IDD-lymphomas and is a part of the three-part nomenclature [3]. EBV-driven oncogenesis induces latent viral gene expression that promotes genomic instability, immune evasion, and resistance to apoptosis [3,21].

CNS IDD-lymphoma is an AIDS-defining malignancy that typically appears in the late stages of HIV infection, with declining incidence in the highly active antiretroviral therapy era (HAART). It primarily affects patients with advanced HIV disease, particularly when CD4+ T-cell counts fall below 50 cells/mm^3^ and viral load is high. The incidence is markedly lower in patients on HAART with effective viral suppression and immune recovery [3].

**Figure 7:**
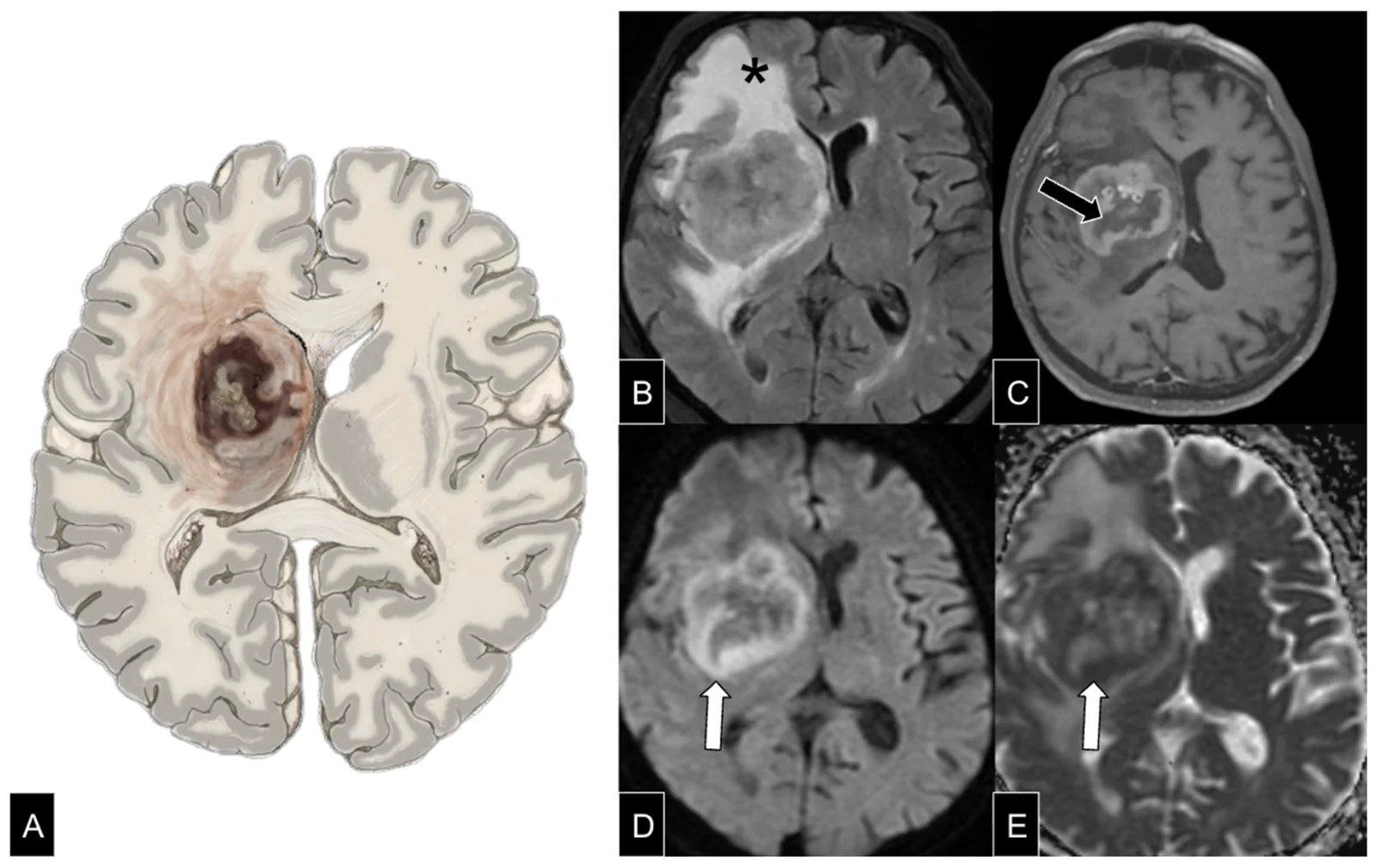
Lymphoma arising in an immune deficiency/dysregulation setting. Axial schematic illustration (**A**) of large B-cell lymphoma a patient living with HIV. Axial FLAIR (**B**), contrast-enhanced T1 weighted MRI (**C**), DWI (**D**) and ADC map (**E**) showing heterogeneous enhancement suggesting necrosis (black arrow), peripheral restricted diffusion (white arrow) and extensive perilesional edema with mass effect (asterisk).

On imaging, heterogeneous enhancement is among the most frequent patterns, observed in approximately 81% of cases [22]. This reflects the lesion’s structural complexity and necrotic composition, with varying proportions of viable tumor tissue, necrosis, and inflammatory infiltrates [2]. Edema and mass effect are common. In immunocompromised patients such as those with HIV, lesions tend to be multiple, larger (≥4 cm), and typically involve the basal ganglia and corpus callosum (Figure S1). Central necrosis and spontaneous hemorrhage often produce irregular or ring-like contrast enhancement. Peripheral restricted diffusion, found in about 71% of cases (Figure 7), is characteristic and reflects the tumor’s aggressive pathology and necrotic core [22].

CNS IDD-lymphoma also occurs in post-transplant recipients as post-transplant lymphoproliferative disorder (PTLD), affecting up to 2% of patients who have undergone solid organ or bone marrow transplantation [23]. This subtype spans a spectrum from benign lymphoid proliferations to aggressive lymphomas, all now grouped under the IDD-lymphoma umbrella [3].

CNS IDD-lymphomas in the post-transplant setting show no sex predilection and are more frequent in children than adults. They exhibit a bimodal incidence peak approximately one year and four to five years post-transplant [24]. A small cohort study found that CNSL occurs more often in recipients of allogeneic stem cell transplants than in solid organ recipients, likely due to more prolonged and intensive immunosuppression [25]. Imaging typically reveals multifocal lesions with ring enhancement, central necrosis, and vasogenic edema. These lesions often involve the basal ganglia, have ill-defined margins, and can mimic other IDD-lymphomas [23] (Figure S2).

### Differential Diagnosis—Advanced and Functional Imaging

DWI plays a critical role in differentiating IDD-lymphoma from other intracranial infections. Tubercular and pyogenic brain abscesses often demonstrate central restricted diffusion due to viscous necrotic material. In contrast, fungal abscesses show peripheral diffusion restriction and may exhibit irregular crenated walls or non–contrast-enhancing intracavitary projections, often with concurrent extracranial involvement [26].

Another DWI feature helps distinguish IDD-lymphoma from its principal mimic in immunosuppressed patients: neurotoxoplasmosis. Neurotoxoplasmosis lesions typically demonstrate central facilitated diffusion due to the low viscosity of internal contents, with higher ADC values compared with lymphomas [27]. In the absence of a tissue diagnosis, short-term follow-up may provide important diagnostic clues, particularly in immunocompromised patients. The *eccentric target sign*, seen on post-contrast T1-weighted images as an enhancing ring with a small eccentric nodule, is a specific finding of neurotoxoplasmosis and is reported in up to 30% of cases (Figure S3) [28].

FDG-PET is a valuable tool for distinguishing cerebral toxoplasmosis from CNSL in an IDD setting when MRI findings are inconclusive. The method leverages the fact that lymphoma demonstrates markedly increased glucose metabolism, whereas toxoplasmosis typically shows reduced or normal uptake relative to surrounding brain tissue. Semiquantitative analyses using standardized uptake values (SUVs) or lesion-to-contralateral brain ratios consistently show higher FDG uptake in lymphoma (SUV ratios ≈ 1.7–3.1) compared with toxoplasmosis (SUV ratios ≈ 0.3–0.7) [29].

Moreover, delayed or dual-time-point FDG-PET further improves diagnostic accuracy: malignant lesions demonstrate progressive radiotracer accumulation over time, while infectious or inflammatory lesions do not. Consequently, FDG-PET—especially with delayed imaging—serves as a valuable noninvasive adjunct to MRI, helping differentiate CNSL from toxoplasmosis and guiding management decisions without immediate need for biopsy [30].

## 4. Intravascular Large B-Cell Lymphoma (IVL)

Intravascular large B-cell lymphoma (IVL) has undergone no major terminological changes in recent WHO classifications. It is a rare and aggressive subtype of extranodal LBCL, defined by the proliferation of neoplastic lymphoid cells within small blood vessels, most commonly affecting the CNS and skin. The median age at diagnosis is approximately 64 years, with no gender predilection. Most cases are of B-cell origin (88%), while 6% and 2% are of T-cell and NK-cell origin, respectively [31].

Unlike other B-cell lymphomas, IVL is confined to the lumina of small- to medium-sized vessels, although minor parenchymal extension may occur. Clinical presentation often includes rapidly progressive and unexplained neurological deficits, frequently mimicking stroke or CNS vasculitis [31].

**Figure 8:**
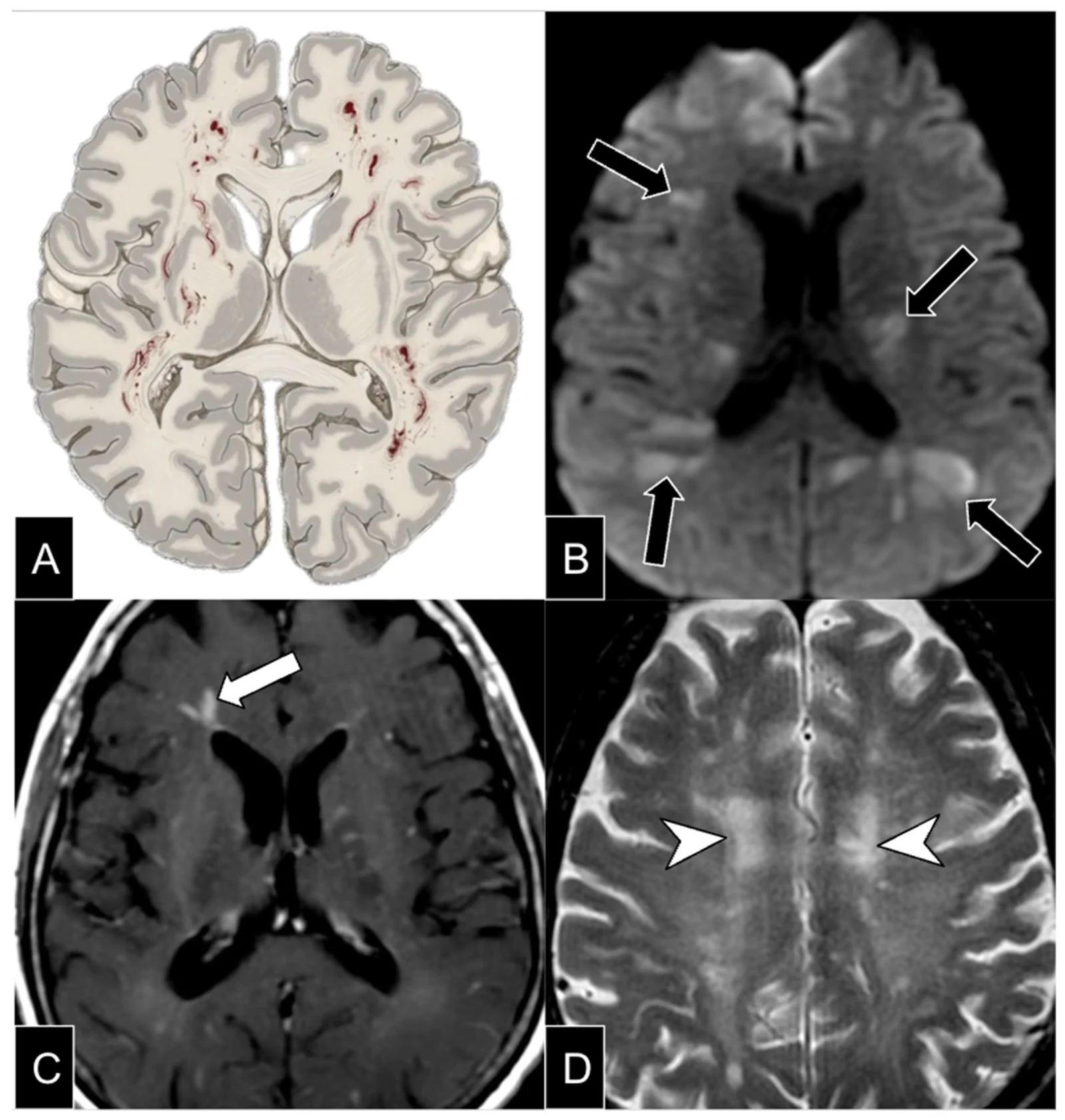
Intravascular lymphoma. Schematic representation (**A**) in a patient showing the proliferation of malignant cells in small and medium-sized blood vessels in the brain. Axial MR DWI (**B**), contrast-enhanced T1 (**C**), and T2 (**D**) demonstrate ischemic lesions with infarct-like areas on DWI (black arrows), with diffuse perivascular enhancement, some showing a tendency toward nodular expansile lesion formation (white arrows), and patchy ill-defined areas of diffuse hyperintense infiltration (arrowheads).

Neuroimaging typically reveals multifocal, bilateral, and asymmetric T2-weighted hyperintensities, predominantly involving deep and subcortical white matter [32]. Lesions frequently demonstrate restricted diffusion on DWI, reflecting cytotoxic edema secondary to vascular occlusion by malignant lymphoid cells. Unlike parenchymal tumors, IVL usually lacks a well-defined mass effect or substantial perilesional edema. Contrast-enhanced MRI may show punctate, patchy, or linear enhancement, typically within affected vessels rather than as nodular or ring-enhancing lesions (Figure 8) [31,32]. Extravascular extension has been described, occasionally forming mass-like lesions resembling parenchymal CNS lymphoma [31].

SWI abnormalities vary from numerous subcortical microhemorrhages and lobar macrohemorrhage to isolated punctate foci of microbleeding. These changes, seen in both supra- and infratentorial regions, may be associated with T2 hyperintensity and/or restricted diffusion, representing secondary hemorrhagic infarctions due to vessel wall injury from intraluminal tumor infiltration [33].

Leptomeningeal involvement, a characteristic feature, appears as diffuse or focal enhancement on post-contrast T1-weighted imaging. This can be subtle and may require high-resolution MRI for detection. IVL-related vascular pathology often produces a “stroke-mimic” pattern, with infarct-like lesions that do not correspond to typical arterial territories, helping differentiate IVL from embolic or thrombotic stroke [31,32].

MR perfusion imaging may show reduced cerebral blood volume (CBV) in affected regions, unlike the increased rCBV seen in high-grade gliomas. While fluorodeoxyglucose (FDG) PET-CT can be valuable in systemic IVL, the role in cerebral disease is not yet completely understood. CNS IVL may demonstrate variable uptake, sometimes appearing hypometabolic due to low temporal resolution, or reduced cell count in the vascular lumen [34].

Given its rarity and nonspecific imaging characteristics, IVL should be considered in patients with rapidly progressive neurological deficits and multifocal ischemia-like lesions, particularly in the absence of conventional vascular risk factors [31,32,33,34].

IVL may affect multiple organs, including the CNS, kidneys, liver, lungs, prostate, skin, and thyroid, while typically sparing hematopoietic organs. The disease may follow a relapsing–remitting or progressive course, with lesions at various stages of evolution. Early diagnosis is crucial to improve outcomes; however, brain biopsy establishes the diagnosis in only a minority of cases before death [32].

## 5. Lymphomatoid Granulomatosis

Lymphomatoid granulomatosis (LYG) is an EBV-associated, angiocentric, and angiodestructive B-cell lymphoproliferative disorder that predominantly involves extranodal tissues. It is characterized by atypical large B cells intermixed with numerous reactive T cells. LYG typically arises in individuals without congenital or acquired immune deficiency, aside from age-related immunosenescence. When it occurs in an immunodeficiency context, the 2024 WHO-HAEM5 recommends the term “polymorphic lymphoproliferative disorder, LYG type, EBV+” [3]. Histopathologically, lesions show atypical lymphohistiocytic infiltrates with angiocentric architecture and polymorphic cellular composition [35].

LYG primarily affects middle-aged adults, most commonly between 40 and 60 years, with an approximate 2:1 male predominance [3]. Although respiratory symptoms are absent in 40–70% of patients, pulmonary involvement is nearly universal, whereas primary CNS disease is exceedingly rare, with only 49 reported cases. Secondary CNS dissemination occurs in up to 40% of cases. Neurologic manifestations include cognitive decline, seizures, and motor disturbances [36]. CNS involvement is associated with high early mortality, particularly within two years of diagnosis, reflecting the aggressive nature of this entity and the need for timely intervention [37].

**Figure 9:**
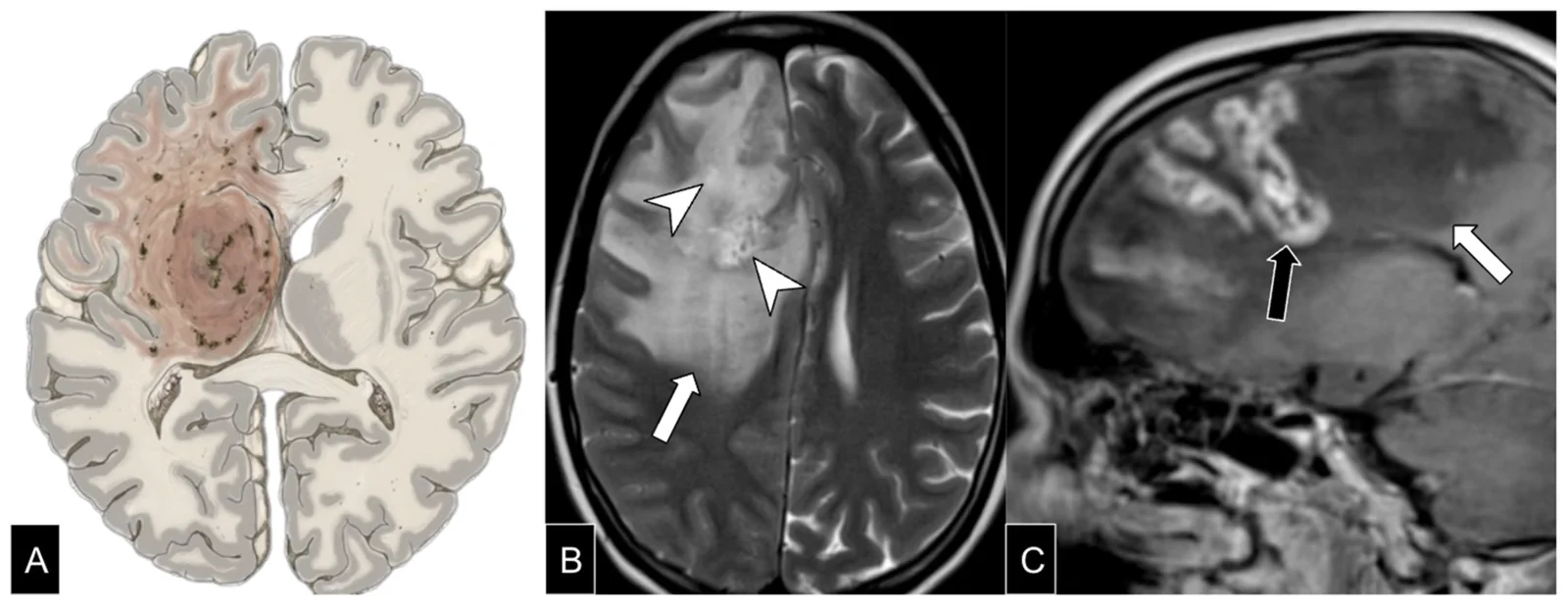
Lymphomatoid granulomatosis. Schematic representation (**A**) in a patient representing a mass-like lesion pattern with necrosis and edema. Axial T2-weighted (**B**) and sagittal contrast-enhanced T1 (**C**) images show multiple hyperintense nodular lesions in the right frontal lobe (arrowheads), with curvilinear contrast enhancement (black arrow), surrounded by an extensive area of edema (white arrows).

Typical MRI findings consist of multiple small enhancing nodules in subependymal or ependymal regions and along vascular structures. These nodules are often isointense on T2-weighted images, show restricted diffusion, and demonstrate nodular or linear enhancement along perivascular spaces [36,37] (Figure 9).

FDG-PET typically demonstrates low metabolic activity, whereas methionine PET shows high uptake. This mismatch has been proposed as a characteristic metabolic pattern, though necrotic or inflammatory lesions remain differential considerations. MRS may reveal decreased NAA, indicating neuronal loss, and increased metabolites suggestive of proliferative activity [36].

Because LYG shares imaging and clinical features with a wide range of other disorders, its diagnosis can be particularly difficult. Both focal and diffuse intracranial processes must be considered in the differential, including IP-CNSL, primary CNS vasculitis, malignant glioma, metastatic neoplasms, and multiple sclerosis, among others [36,37].

## 6. Lymphomatosis Cerebri

Lymphomatosis cerebri (LC) is a rare manifestation of CNSL. It is not recognized as a distinct entity by the WHO but represents a clinical–radiologic presentation of various lymphoma subtypes, most commonly LBCL, though it may also occur in T-cell lymphomas [38]. The term was first introduced by Bakshi et al., who described two patients with rapidly progressive dementia and diffuse cerebral infiltration by lymphoma cells [39].

LC typically affects individuals in their sixth to seventh decades of life, with a slight male predominance, mirroring the epidemiology of DLBCL. Clinical features include subacute cognitive decline, gait disturbance, and behavioral changes. CSF analysis shows elevated protein levels in most cases and pleocytosis in approximately half [40].

**Figure 10:**
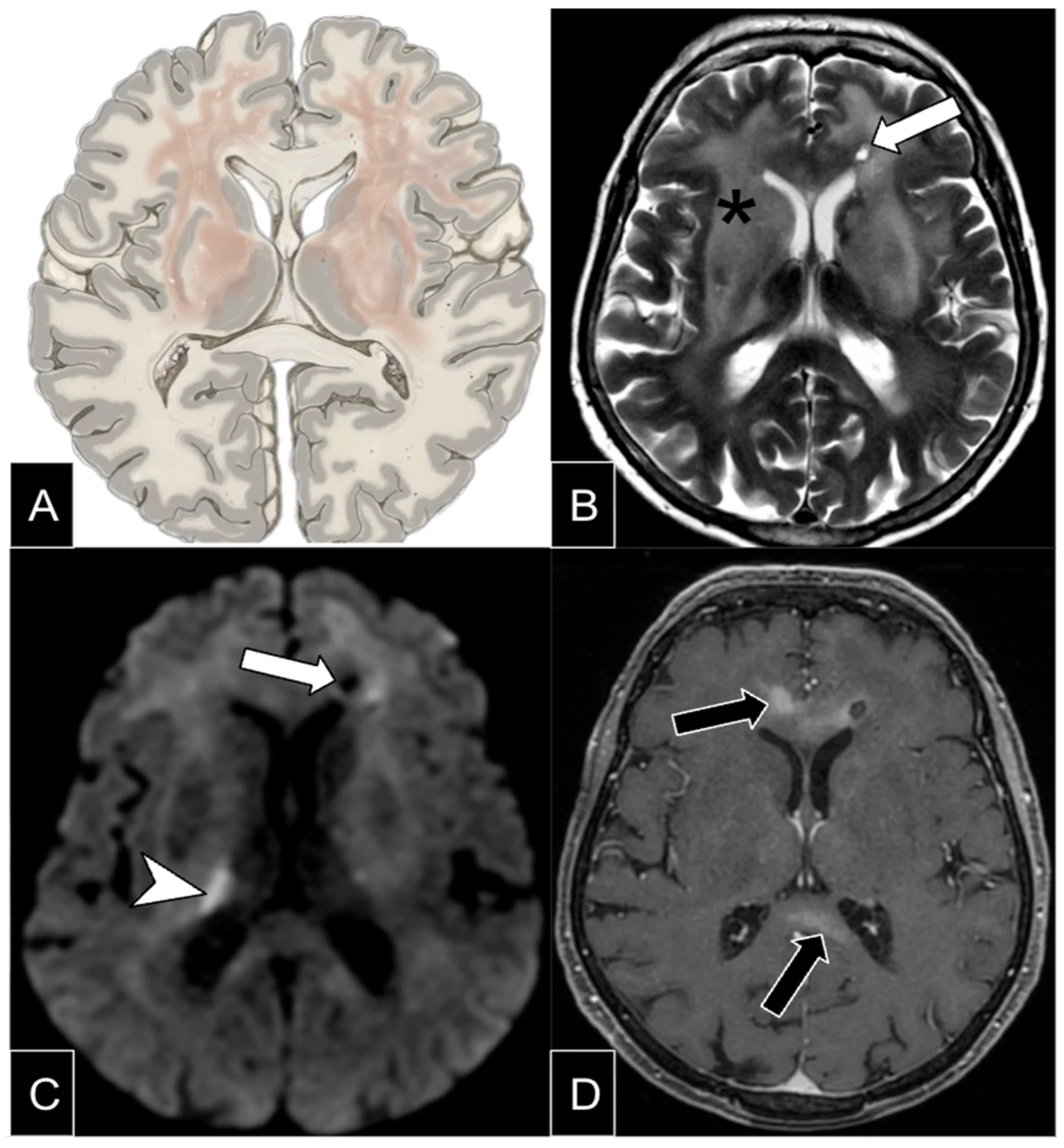
Lymphomatosis cerebri. Schematic representation (**A**) and axial T2-weighted images (**B**) in a patient show diffuse infiltrative hyperintensity involving the deep white matter and basal ganglia in both cerebral hemispheres (asterisks). Axial contrast-enhanced T1-weighted (**C**) and DWI (**D**) show discrete patchy enhancement (black arrows) and some foci of restricted diffusion (arrowhead). Also note the biopsy site in the left frontal lobe (white arrows).

On MRI, LC appears as diffuse T2-weighted and FLAIR hyperintensity with minimal or absent contrast enhancement, predominantly involving the supratentorial brain. Bilateral, diffuse hemispheric involvement is characteristic, while infratentorial disease is less frequent and spinal involvement rare (Figure 10) [40]. Diffusion restriction is common and aids differentiation from other infiltrative disorders. MRS typically shows increased choline and lipid peaks with decreased NAA and creatine levels [40]. Okazaki et al. proposed arterial spin labeling to optimize biopsy targeting by distinguishing tumor-rich regions from vasogenic edema, thereby improving diagnostic yield [41].

LC carries a dismal prognosis, with median survival of about four months—worse than that of conventional DLBCL [40]. Follow-up imaging often demonstrates the evolution of patchy enhancement in previously nonenhancing lesions and mass-like enhancement in those with prior patchy enhancement, reflecting progressive blood–brain barrier disruption [38,40].

The differential diagnosis primarily includes gliomatosis cerebri (GC), which, like LC, is no longer considered a distinct WHO entity but a radiologic pattern of diffuse glioma infiltration. Seizures are more frequent in GC [40]. Other differential considerations include progressive multifocal leukoencephalopathy, small-vessel vasculopathy, toxic–metabolic disturbances, and demyelinating diseases [40].

## 7. Secondary Lymphoma of the CNS

Secondary CNS lymphoma (SCNSL) is defined as CNS involvement either at the initial diagnosis of systemic lymphoma (de novo SCNSL) or at relapse, when it may occur with systemic disease or as isolated CNS involvement [42].

SCNSL is uncommon, affecting <1% of indolent lymphomas and <5% of aggressive lymphomas with routine CNS prophylaxis. Without prophylaxis, it develops in up to 50% of patients with Burkitt lymphoma or AIDS-related lymphomas. LBCL is the most frequent histologic subtype [43].

Although earlier studies suggested leptomeningeal involvement in up to two-thirds of cases, recent data indicate that parenchymal disease predominates (40–60%), followed by leptomeningeal (20–30%) and combined involvement (10%) [42]. Clinical manifestations depend on the affected regions and include motor deficits, headache, cranial neuropathies, and cognitive or behavioral changes [44].

**Figure 11:**
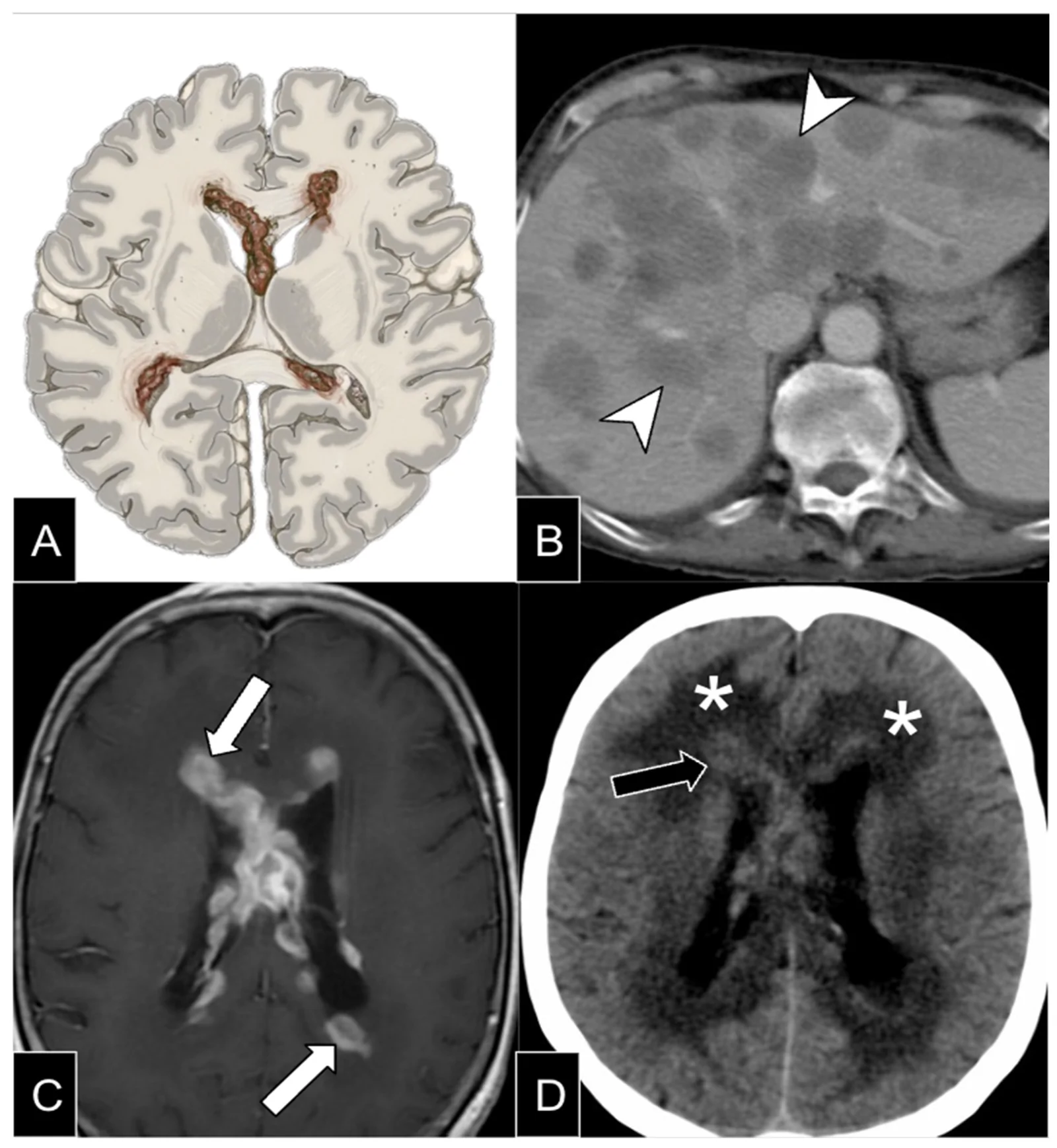
Systemic lymphoma with secondary CNS involvement. Schematic drawing (**A**) and axial CT image (**B**) shows diffuse nodular liver involvement (arrowheads). Axial contrast enhanced T1-weighted MR image (**C**) shows CNS involvement with extensive periventricular confluent nodular lesions with strong contrast enhancement in the periventricular white matter (white arrows). Axial CT (**D**) shows hyperdense periventricular lesions (black arrow) and also hypodensity in the surrounding white matter due to vasogenic edema (asterisk).

MRI is the modality of choice for CNS assessment. Lesions are typically T2-hypointense with restricted diffusion and homogeneous enhancement (Figure 11). They are often multiple, predominantly supratentorial or both supra- and infratentorial, with nonenhancing lesions being rare. MRI findings alone cannot reliably differentiate primary from secondary CNS lymphoma. Comprehensive systemic evaluation—including blood and CSF studies, whole-body PET/CT, spinal MRI, fundoscopy, and testicular ultrasound (if PET/CT is unavailable)—is therefore essential [44].

**Figure 12:**
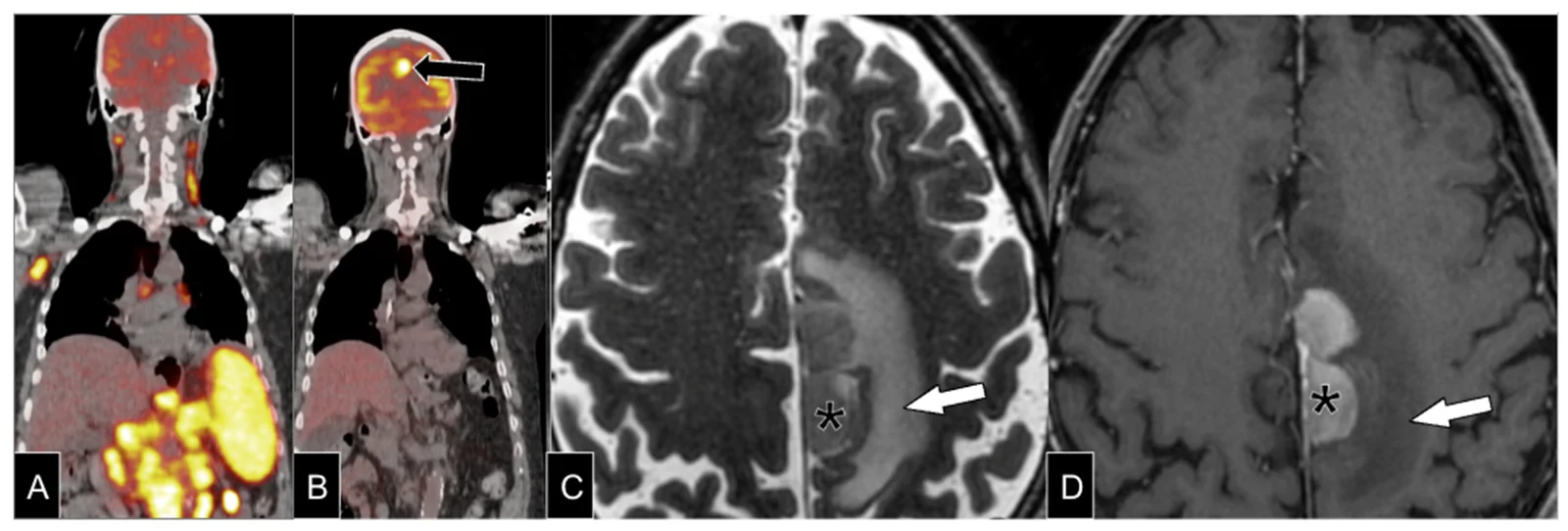
Systemic lymphoma and CNS relapse. Baseline FDG-PET/CT (**A**) shows increased uptake in multiple lymph node chains and spleen, without CNS involvement. Six-month follow-up post-treatment FDG-PET/CT (**B**) shows remission of abdominal disease, with a new brain lesion (black arrow). Axial T2-weighted (**C**) and contrast enhanced T1-weighted (**D**) MR images show a parenchymatous expansive lesion (asterisks) with low T2 signal intensity and homogeneous contrast enhancement. Also note prominent perilesional edema (white arrow).

Most SCNSL cases arise at relapse rather than de novo, with isolated CNS disease predominating (Figure 12) [42]. Relapses typically occur within the first year, and de novo cases generally have better overall survival [42,44].

## 8. Mucosa-Associated Lymphoid Tissue (MALT) Lymphoma of the Dura

Mucosa-associated lymphoid tissue (MALT) lymphoma is an indolent extranodal non-Hodgkin lymphoma derived from mature B cells of the marginal zone. Although the stomach is the most commonly affected site, MALT lymphoma can arise in virtually any organ and, rarely, involve the dura mater intracranially. Because of its rarity, the true incidence is uncertain, with just over 100 reported cases. A female predominance is observed, with a median age at diagnosis of approximately 65 years [45].

Lesions typically present as slow-growing, homogeneous extra-axial dural masses. On CT, they are usually hyperdense, while on MRI they appear hypo- to isointense on T1-weighted and hypointense on T2-weighted images, often showing restricted diffusion and homogeneous enhancement [2,45].

Owing to the absence of a specific imaging signature, MALT lymphoma may mimic meningioma. However, features such as bone preservation or subtle permeative changes from tumor spread through Haversian canals (Figure S4) help distinguish it. In contrast, meningiomas often demonstrate hyperostosis, whereas plasmacytomas and metastases typically exhibit aggressive lytic bone destruction [46].

## 9. T-Cell and Natural Killer Lymphoma

In the 2021 WHO-CNS, T-cell and NK/T-cell lymphomas are included among miscellaneous rare CNS lymphomas, along with anaplastic large cell lymphomas (ALK+/ALK−) and low-grade B-cell CNS lymphomas. These entities display variable imaging features that can resemble edema, glial tumors, or infectious and inflammatory processes [2,3]. T-cell lymphomas are aggressive neoplasms with poor prognosis, accounting for approximately 2% of primary CNS lymphomas and 0.4% of all non-Hodgkin lymphomas (NHL). Certain subtypes are EBV-associated, more commonly involving the paranasal region, skin, and gastrointestinal tract; CNS involvement is exceedingly rare [47]. Reported cases appear as lobar masses, though leptomeningeal and cauda equina lesions have been described [47,48] (Figure S5).

## 10. Treatment and Related Complications

Because of the high chemosensitivity of CNSL, induction therapy typically relies on high-dose methotrexate, often combined with rituximab in CD20-positive cases, and may include agents such as procarbazine, vincristine, or temozolomide. Consolidation strategies usually involve high-dose chemotherapy followed by autologous stem cell transplantation. For patients ineligible for intensive therapy—particularly those over 65 years—non-myeloablative chemotherapy or reduced-dose whole-brain radiotherapy are considered appropriate alternatives [49]. Dural MALT lymphoma represents an exception, as treatment generally consists of surgical resection followed by localized radiotherapy [50].

MRI plays a central role not only in diagnosis but also in monitoring treatment response. Dynamic MRI changes, including variations in lesion size and enhancement, provide valuable information on therapeutic efficacy. These imaging alterations often correlate with clinical improvement, supporting MRI as a key tool for response assessment [50].

In PCNS-LBCL, imaging biomarkers can predict treatment response and prognosis. FDG-PET, particularly after two cycles of high-dose methotrexate, demonstrates superior predictive accuracy compared with MRI. PET negativity at this stage strongly correlates with complete response and prolonged progression-free survival, suggesting that early metabolic changes precede morphological regression and serve as a robust indicator of therapeutic efficacy [51].

Therapeutic regimens combining corticosteroids and chemotherapy may result in neurotoxicity—such as methotrexate-associated leukoencephalopathy (MTX-LE). Methylenetetrahydrofolate reductase (MTHFR) gene polymorphisms may indicate high risk of MTX-LE, with a significant impact on prognosis and mortality of affected patients (Figure S6). Treatment-related immunosuppression predisposes patients to opportunistic infections, including JC virus, herpesviruses, fungi, *Listeria monocytogenes*, and *Toxoplasma gondii* [52,53].

## 11. Future Directions and Conclusions

Future research in neuroimaging for CNS lymphoproliferative disorders should focus on advancing multiparametric MRI and radiomics to improve diagnostic precision, enable more accurate subtype differentiation, and enhance understanding of underlying tumor biology. Combining advanced MRI techniques has shown potential to better distinguish CNS lymphoma from other brain tumors and to non-invasively identify biologically relevant subtypes. Emerging radiomics and habitat-based models offer promising ways to capture intratumoral heterogeneity and predict molecular and prognostic features without the need for invasive procedures. To achieve meaningful clinical translation, future studies should prioritize large, multicenter validation, standardized imaging protocols, longitudinal imaging–genomic correlations, and the integration of interpretable machine-learning tools.

CNSL comprises a rare, heterogeneous group of diseases affecting both immunocompetent and immunodeficient individuals, with or without EBV association. Biopsy remains essential for diagnosis, but conventional and advanced imaging play a critical role in suggesting diagnosis, guiding differential considerations, and monitoring therapy. Radiologists must recognize the varied imaging patterns of these disorders to contribute effectively to multidisciplinary neuro-oncologic management.

## Figures and Tables

**Table 1:** Main clinicoradiological characteristics of CNS lymphoid disorders. Note: LBCL-IP = Large B-cell lymphoma of immune-privileged sites, IDD-lymphomas = lymphomas arising in immune deficiency/dysregulation, EBV = Epstein–Barr Virus, HHV-8 = human herpes virus 8, GFAP = glial fibrillary acidic protein, SWI = susceptibility-weighted imaging, MALT = mucosa-associated lymphoid tissue.

	Epidemiology	Imaging Features	Diagnostic Criteria	Imaging Differential Diagnosis	Prognosis
LBCL-IP	1–2% of the Non-Hodgkin Lymphomas 2–3% of Brain Tumors 0.5–1 cases per 100,000 person-years	Periventricular, restricted diffusion, low T2, homogeneous enhancement, minor edema	**Essential**: LBCL confined to the CNS, vitreoretina or testis, exclusion of secondary involvement and IDD context. **Desirable**: MUM1+, BCL6+, CD10−, EBV−, and MYD88 and CD79B mutations.	Glioblastoma: higher ADC, higher perfusion, heterogeneous enhancement Metastases: multiple corticosubcortical, significant edema Inflammatory/infectious: ring enhancement	5-year survival rate: 30–40% Median survival: between 2 and 5 years
IDD-lymphomas	Linked to EBV or HHV-8; IDD context: AIDS, post-transplant, immunotherapy, immunosuppressive agents.	Solitary/multifocal heterogeneous ring-enhancing lesions, peripheral restricted diffusion	**Essential**: IDD context and specific diagnostic criteria for each pathology (e.g., LBCL). **Desirable**: tissue detection of EBV or HHV8.	Toxoplasmosis: eccentric target sign Fungal abscesses: SWI double halo sign Vasculitis: multifocal ischemic lesions	High mortality rate, Influenced by degree of immunosuppression
Intravascular Lymphoma	Rare subtype. Median age: 70 years. Possible genetic predisposition in Asian and European countries.	Multifocal T2 hyperintensities, mass-like lesions, infarct-like injuries, perivascular enhancement.	**Essential**: Lymphoid cells restricted to capillaries, CD20+, CD79a+. **Desirable**: negative EBV and HHV-8 in tissue.	Vasculitis: white matter lesions, infarcts and hemorrhage GFAP astrocytopathy: cauda equina enhancement/pleocytosis Stroke mimic patterns: lack of vascular territory conformity	Rapid progression, increasing antemortem diagnosis. Median survival: 2–8 months.
LYG	<500 cases reported Median age: 4th to 6th decades. M:F ratio: 2:1	Diffuse parenchymal infiltration, heterogeneous expansile lesion, edema, nodular or linear enhancement	**Essential**: Extranodal disease with polymorphous angiocentric lymphoid infiltrate, small-to-medium-sized vessel involvement by large B-cells (EBV+), exclusion of IDD setting other than immunosenescence	LBCL/IVL: biopsy needed GPA: paranasal sinus, kidney Metastasis: corticosubcortical, higher DWI Vasculitis: enhancement along vessels, hemorrhage Neurosarcoidosis: nodular/linear	Variable prognosis High mortality within two years
Lymphomatosis Cerebri	Extremely rare manifestation, typically 6th/7th decades	Diffuse T2/FLAIR hyperintensity affecting at least 3 cerebral lobes Minimal contrast enhancement	Diffuse brain lymphomatous infiltrate	Gliomatosis cerebri: similar infiltrative pattern but frequent seizures, biopsy needed LEMP: viral pathology	Median survival time of 4 months
Secondary Lymphoma of the CNS	5–29% of patients with systemic LBCL <1% of indolent lymphomas and <5% of aggressive lymphomas	Hyperintense on T2, restricted diffusion, homogeneous enhancement 2/3 leptomeningeal 1/3 parenchymal	Systemic workup mandatory, higher risk in LBCL, Burkitt’s and lymphoblastic lymphoma.	Metastases: systemic origin, high PWI Sarcoidosis: Suprasellar/pulmonary involvement Fungal abscesses: immunosuppression, peripheral diffusion restriction	Survival between 2 and 6 months Recurrence in 50% of cases with better prognosis
MALT Lymphoma of the Dura	Rare subtype of extranodal B-cell marginal zone lymphoma Median age 65 years, female predominance Related to autoimmune diseases.	Dural mass without adjacent bone erosion, hypo- to isointense on T1, hypointense on T2	**Essential**: Atypical lymphocytes resembling reactive, B-cell markers, exclusion of other B-cell lymphomas.	Meningioma: hyperostosis Plasmacytomas: more aggressive bone destruction	Favorable compared to LBCL with appropriate treatment
T-Cell and Natural Killer Cell Lymphoma	10–15% of all non-Hodgkin lymphomas Aggressive, poor prognosis, 2% of CNSL	Diverse patterns (lobar masses, pituitary lesions, leptomeningeal disease) Similar to LBCL but with more necrosis	T-cell markers (CD3, CD4, CD8, CD56+)	B-Cell Lymphomas: biopsy needed Vasculitis: white matter lesions, infarcts and hemorrhage Gliomas: high perfusion Inflammatory/infectious: leptomeningeal or mass lesions	More aggressive behavior and poorer clinical outcomes than B-cell lymphomas

## Data Availability

No new data were created or analyzed in this study. Data sharing is not applicable to this article.

## Supplementary Materials

The following supporting information can be downloaded at https://www.mdpi.com/article/10.3390/diagnostics16182932/s1, Figure S1. Lymphoma arising in immune deficiency/dysregulation (IDD-lymphoma). Axial FLAIR (A) and contrast-enhanced T1-weighted MRI (B) display a single subcortical mass lesion (black arrow) in a patient recently diagnosed with HIV, with thick contrast enhancement (arrowhead) and vasogenic edema (asterisk). Follow-up axial FLAIR (C) and contrast-enhanced T1-weighted MRI (D) after two months with appropriate antiparasitic treatment, demonstrating lesion (black arrows) and edema progression (asterisk), with new thalamic and contralateral lesions (white arrows), related to biopsy-proven IDD-lymphoma. Figure S2. Lymphoma arising in post-bone-marrow-transplant setting. Axial FLAIR (A) and ADC (B) images in a patient one year after bone marrow transplant presenting with a well-defined and homogenous enhancing lesion affecting the left temporal lobe (black arrow), with low ADC values (white arrow) and vasogenic edema. Biopsy of concurrent lung mass confirmed a post-transplant lymphoproliferative disorder. Figure S3. Differential diagnosis of lymphomas arising in immune deficiency/dysregulation (IDD-lymphoma). (A) Axial contrast-enhanced T1-weighted MRI showing a periventricular toxoplasmosis lesion (white arrow) in a patient living with HIV (PLWH), mimicking IDD-lymphoma imaging features. (B) Coronal contrast-enhanced T1-weighted MRI shows subcortical neurotoxoplasmosis lesions with eccentric target sign (yellow arrow) in a patient PLWH. (C) Susceptibility-weighted imaging of a patient after bone marrow transplant with CNS aspergillosis showing the double rim sign (black arrow). Figure S4. Mucosal-associated lymphoid tissue (MALT) lymphoma of the dura. (A) Schematic representation of MALT lymphoma of the dura mater. (B) Coronal MRI of a patient with a solid extra-axial mass in the right frontoparietal high convexity extending extracranially (white arrow). (C) Axial CT shows subtle permeative skull changes out of proportion to the lesion’s apparent size (black arrow). (D) Metastatic breast cancer in a patient with destructive lytic pattern of the skull (arrowheads) for comparison purposes. Figure S5. T-cell lymphoma. (A) Schematic representation. (B) T2-weighted image in a patient shows focal lesion with high signal intensity and adjacent vasogenic edema (black arrow). (C) T2-weighted gradient-echo showing microhemorrhagic foci. (D) Post-contrast T1-weighted image demonstrates irregular and heterogeneous enhancement. Figure S6. Treatment evolution in large B-cell lymphoma of immune-privileged sites. Serial axial T1-contrast-enhanced MRI (A-C) in a patient demonstrating mass lesions (white arrows) centered in frontal lobes. (A) Baseline scan shows heterogeneously enhancing lesions with associated mass effect. (B) After 9 months of radiochemotherapy, there is marked reduction in lesion volume and contrast enhancement. (C) Follow-up study 2 years post-treatment demonstrates complete radiologic remission. (D) Axial FLAIR image from a different patient illustrates methotrexate-induced leukoencephalopathy, with confluent periventricular and deep white matter hyperintensities (black arrows).

## References

[1] Miller K.D., Ostrom Q.T., Kruchko C., Patil N., Tihan T., Cioffi G., Fuchs H.E., Waite K.A., Jemal A., Siegel R.L. (2021). Brain and other central nervous system tumor statistics, 2021. CA Cancer J. Clin..

[2] WHO Classification of Tumours Editorial Board (2021). World Health Organization Classification of Tumours of the Central Nervous System.

[3] WHO Classification of Tumours Editorial Board (2024). Hematolymphoid Tumors. World Health Organization Classification of Tumours.

[4] Song K.W., Issa S., Batchelor T. (2021). Primary central nervous system lymphoma: Epidemiology and clinical presentation. Ann. Lymphoma.

[5] Nguyen-Them L., Alentorn A., Ahle G., Soussain C., Mathon B., Le Garff Tavernier M., Houillier C., Hoang-Xuan K. (2023). CSF biomarkers in primary CNS lymphoma. Rev. Neurol..

[6] Tang D., Chen Y., Shi Y., Tao H., Tao S., Zhang Q., Ding B., He Z., Yu L., Wang C. (2022). Epidemiologic Characteristics, Prognostic Factors, and Treatment Outcomes in Primary Central Nervous System Lymphoma: A SEER-Based Study. Front. Oncol..

[7] Haldorsen I.S., Espeland A., Larsson E.M. (2011). Central nervous system lymphoma: Characteristic findings on traditional and advanced imaging. AJNR Am. J. Neuroradiol..

[8] Gómez Roselló E., Quiles Granado A.M., Laguillo Sala G., Pedraza Gutiérrez S. (2018). Primary central nervous system lymphoma in immunocompetent patients: Spectrum of findings and differential characteristics. Radiol. (Engl. Ed.).

[9] Johnson B., Fram E., Johnson P., Jacobowitz R. (1997). The variable MR appearance of primary lymphoma of the central nervous system: Comparison with histopathologic features. AJNR Am. J. Neuroradiol..

[10] Velasco R., Mercadal S., Vidal N., Alañá M., Barceló M.I., Ibáñez-Juliá M.J., Bobillo S., Caldú Agud R., García Molina E., Martínez P. (2020). Diagnostic delay and outcome in immunocompetent patients with primary central nervous system lymphoma in Spain: A multicentric study. J. Neurooncol..

[11] Xiang C., Chen Q., Zha Y. (2018). Specific Features of Primary Central Nervous System Lymphoma in Comparison with Glioblastoma on Conventional MRI. Iran. J. Radiol..

[12] Cetinoglu Y.K., Sinci K.A., Horoz M., Gelal F. (2025). Qualitative MRI features in the differentiation between primary and secondary CNS lymphoma. Neuroradiology.

[13] Patra A., Jasper A., Vanjare H., Chacko G., Susheel S., Sivadasan A., Hephzibah J., Mannam P. (2021). Diffuse Infiltrative Non-mass-like Brain Parenchymal Lesions on MRI: Differentiating Lymphomatosis Cerebri from its Mimics. J. Clin. Imaging Sci..

[14] Makino K., Hirai T., Nakamura H., Kuroda J., Shinojima N., Uetani H., Kitajima M., Yano S. (2017). Differentiating Between Primary Central Nervous System Lymphomas and Glioblastomas: Combined Use of Perfusion-Weighted and Diffusion-Weighted Magnetic Resonance Imaging. World Neurosurg..

[15] Mora P., Majós C., Castañer S., Sánchez J.J., Gabarrós A., Muntané A., Aguilera C., Arús C. (2014). (1)H-MRS is useful to reinforce the suspicion of primary central nervous system lymphoma prior to surgery. Eur. Radiol..

[16] Mansour A., Qandeel M., Abdel-Razeq H., Abu Ali H.A. (2014). MR imaging features of intracranial primary CNS lymphoma in immune competent patients. Cancer Imaging.

[17] Ambady P., Hu L.S., Politi L.S., Anzalone N., Barajas R.F. (2021). Primary central nervous system lymphoma: Advances in MRI and PET imaging. Ann. Lymphoma.

[18] Surendra K.L., Patwari S., Agrawal S., Chadaga H., Nagadi A. (2020). Percentage signal intensity recovery: A step ahead of rCBV in DSC MR perfusion imaging for the differentiation of common neoplasms of brain. Indian J. Cancer.

[19] Xing Z., You R., Li J., Liu Y., Cao D. (2014). Differentiation of Primary Central Nervous System Lymphomas from High-Grade Gliomas by rCBV and Percentage of Signal Intensity Recovery Derived from Dynamic Susceptibility-Weighted Contrast-Enhanced Perfusion MR Imaging. Clin. Neuroradiol..

[20] Valles F.E., Perez-Valles C.L., Regalado S., Barajas R.F., Rubenstein J.L., Cha S. (2013). Combined diffusion and perfusion MR imaging as biomarkers of prognosis in immunocompetent patients with primary central nervous system lymphoma. AJNR Am. J. Neuroradiol..

[21] Yin H., Qu J., Peng Q., Gan R. (2019). Molecular mechanisms of EBV-driven cell cycle progression and oncogenesis. Med. Microbiol. Immunol..

[22] Reis F., Schwingel R., do Nascimento F.B.P. (2013). Linfoma do sistema nervoso central: Ensaio iconográfico. Radiol. Bras..

[23] Penn I. (2000). Post-transplant malignancy: The Role of immunosuppression. Drug Saf..

[24] White M.L., Moore D.W., Zhang Y., Mark K.D., Greiner T.C., Bierman P.J. (2019). Primary central nervous system post-transplant lymphoproliferative disorders: The spectrum of imaging appearances and differential. Insights Imaging.

[25] Taj M.M., Maecker-Kolhoff B., Ling R., Bomken S., Burkhardt B., Chiang A.K.S., Csoka M., Füreder A., Haouy S., Lazic J. (2021). Primary post-transplant lymphoproliferative disorder of the central nervous system: Characteristics, management and outcome in 25 paediatric patients. Br. J. Haematol..

[26] Luthra G., Parihar A., Nath K., Jaiswal S., Prasad K.N., Husain N., Husain M., Singh S., Behari S., Gupta R.K. (2007). Comparative evaluation of fungal, tubercular, and pyogenic brain abscesses with conventional and diffusion MR imaging and proton MR spectroscopy. AJNR Am. J. Neuroradiol..

[27] Camacho D.L., Smith J.K., Castillo M. (2003). Differentiation of toxoplasmosis and lymphoma in AIDS patients by using apparent diffusion coefficients. AJNR Am. J. Neuroradiol..

[28] Kumar G.G., Mahadevan A., Guruprasad A.S., Kovoor J.M., Satishchandra P., Nath A., Ranga U., Shankar S.K. (2010). Eccentric target sign in cerebral toxoplasmosis: Neuropathological correlate to the imaging feature. J. Magn. Reson. Imaging.

[29] Omid-Fard N., Puac-Polanco P., Torres C.H., Hamilton L., Nguyen T.B. (2025). Imaging Features of Immunodeficiency-Associated Primary CNS Lymphoma: A Review. Can. Assoc. Radiol. J..

[30] Marcus C., Feizi P., Hogg J., Summerfield H., Castellani R., Sriwastava S., Marano G.D. (2021). Imaging in Differentiating Cerebral Toxoplasmosis and Primary CNS Lymphoma With Special Focus on FDG PET/CT. AJR Am. J. Roentgenol..

[31] Yamamoto A., Kikuchi Y., Homma K., O’uchi T., Furui S. (2012). Characteristics of intravascular large B-cell lymphoma on cerebral MR imaging. AJNR Am. J. Neuroradiol..

[32] Song D.K., Boulis N.M., McKeever P.E., Quint D.J. (2002). Angiotropic large cell lymphoma with imaging characteristics of CNS vasculitis. AJNR Am. J. Neuroradiol..

[33] Richie M.B., Guterman E.L., Shah M.P., Cha S. (2022). Susceptibility-Weighted Imaging of Intravascular Lymphoma of the Central Nervous System. JAMA Neurol..

[34] Zhao D., Zhang Y., Zhu W., Huo L., Zhou D., Wang W., Wei C., Zhang W. (2024). Distinct FDG PET/CT avidity among newly diagnosed intravascular large B-cell lymphoma patients: A descriptive observational study. Ann. Hematol..

[35] Tateishi U., Terae S., Ogata A., Sawamura Y., Suzuki Y., Abe S., Miyasaka K. (2001). MR imaging of the brain in lymphomatoid granulomatosis. AJNR Am. J. Neuroradiol..

[36] Xiang Y., Liu C., Xue Y., Li S., Sui Y., Li J., Sun Q., Liu X. (2020). Primary Central Nervous System Lymphomatoid Granulomatosis: Systemic Review. Front. Neurol..

[37] Patsalides A.D., Atac G., Hedge U., Janik J., Grant N., Jaffe E.S., Dwyer A., Patronas N.J., Wilson W.H. (2005). Lymphomatoid granulomatosis: Abnormalities of the brain at MR imaging. Radiology.

[38] Izquierdo C., Velasco R., Vidal N., Sánchez J.J., Argyriou A.A., Besora S., Graus F., Bruna J. (2016). Lymphomatosis cerebri: A rare form of primary central nervous system lymphoma. Analysis of 7 cases and systematic review of the literature. Neuro Oncol..

[39] Bakshi R., Mazziotta J.C., Mischel P.S., Jahan R., Seligson D.B., Vinters H.V. (1999). Lymphomatosis cerebri presenting as a rapidly progressive dementia: Clinical, neuroimaging and pathologic findings. Dement. Geriatr. Cogn. Disord..

[40] Fan M., Zhao L., Chen Q., Zhang M., Zhang X., Yang Z., Li S., Song Y. (2023). Clinical and imaging features of lymphomatosis cerebri: Analysis of 8 cases and systematic review of the literature. Clin. Exp. Med..

[41] Okazaki A., Yamasaki T., Kataoka E., Fujihiro M., Kurozumi K. (2024). Clinical Benefits of Arterial Spin-Labeling Magnetic Resonance Imaging for Primary Diffuse Large B-cell Lymphoma of the Central Nervous System Presenting With Lymphomatosis Cerebri: A Case Report. Cureus.

[42] Treiber H., Nilius-Eliliwi V., Seifert N., Vangala D., Wang M., Seidel S., Mika T., Marschner D., Zeremski V., Wurm-Kuczera R. (2023). Treatment Strategies and Prognostic Factors in Secondary Central Nervous System Lymphoma: A Multicenter Study of 124 Patients. Hemasphere.

[43] Malikova H., Burghardtova M., Koubska E., Mandys V., Kozak T., Weichet J. (2018). Secondary central nervous system lymphoma: Spectrum of morphological MRI appearances. Neuropsychiatr. Dis. Treat..

[44] Bobillo S., Khwaja J., Ferreri A.J.M., Cwynarski K. (2023). Prevention and management of secondary central nervous system lymphoma. Haematologica.

[45] Glinka P., Sobstyl M., Rymkiewicz G., Wierzba-Bobrowicz T., Paszkiewicz-Kozik E., Grajkowska W. (2024). Extranodal marginal zone lymphoma of mucosa-associated lymphoid tissue of the dura mimicking meningioma: A case report and literature review. Folia Neuropathol..

[46] Ferguson S.D., Musleh W., Gurbuxani S., Shafizadeh S.F., Lesniak M.S. (2010). Intracranial mucosa-associated lymphoid tissue (MALT) lymphoma. J. Clin. Neurosci..

[47] Nevel K.S., Pentsova E., Daras M. (2019). Clinical presentation, treatment, and outcomes of patients with central nervous system involvement in extranodal natural killer/T-cell lymphoma. Leuk. Lymphoma.

[48] Morita M., Osawa M., Naruse H., Nakamura H. (2009). Primary NK/T-cell lymphoma of the cauda equina: A case report and literature review. Spine.

[49] Ferreri A.J.M., Calimeri T., Cwynarski K., Dietrich J., Grommes C., Hoang-Xuan K., Hu L.S., Illerhaus G., Nayak L., Ponzoni M. (2023). Primary central nervous system lymphoma. Nat. Rev. Dis. Primers.

[50] Kaji F.A., Martinez-Calle N., Sovani V., Fox C.P. (2022). Rare central nervous system lymphomas. Br. J. Haematol..

[51] Rozenblum L., Houillier C., Baptiste A., Soussain C., Edeline V., Naggara P., Soret M., Causse-Lemercier V., Willems L., Choquet S. (2024). Interim FDG-PET improves treatment failure prediction in primary central nervous system lymphoma: An LOC network prospective multicentric study. Neuro-Oncology.

[52] Karschnia P., Kurz S.C., Brastianos P.K., Winter S.F., Gordon A., Jones S., Pisapia M., Nayyar N., Tonn J.C., Batchelor T.T. (2023). Association of MTHFR Polymorphisms with Leukoencephalopathy Risk in Patients with Primary CNS Lymphoma Treated With Methotrexate-Based Regimens. Neurology.

[53] Pruitt A.A. (2021). Central Nervous System Infections in Immunocompromised Patients. Curr. Neurol. Neurosci. Rep..

