# Supplementary material for: Spectrum of Neuroimaging Findings in CNS Lymphoproliferative Disorders
Source: Diagnostics (Basel). 2026 Sep 10;16(18):2932. doi: 10.3390/diagnostics16182932 (PMC13605541; doi:10.3390/diagnostics16182932)
Supplement: Supplementary file 1 [file diagnostics-16-02932-s001.zip › diagnostics-4509094-supplementary.pdf]

SUPPLEMENTAL FIGURES

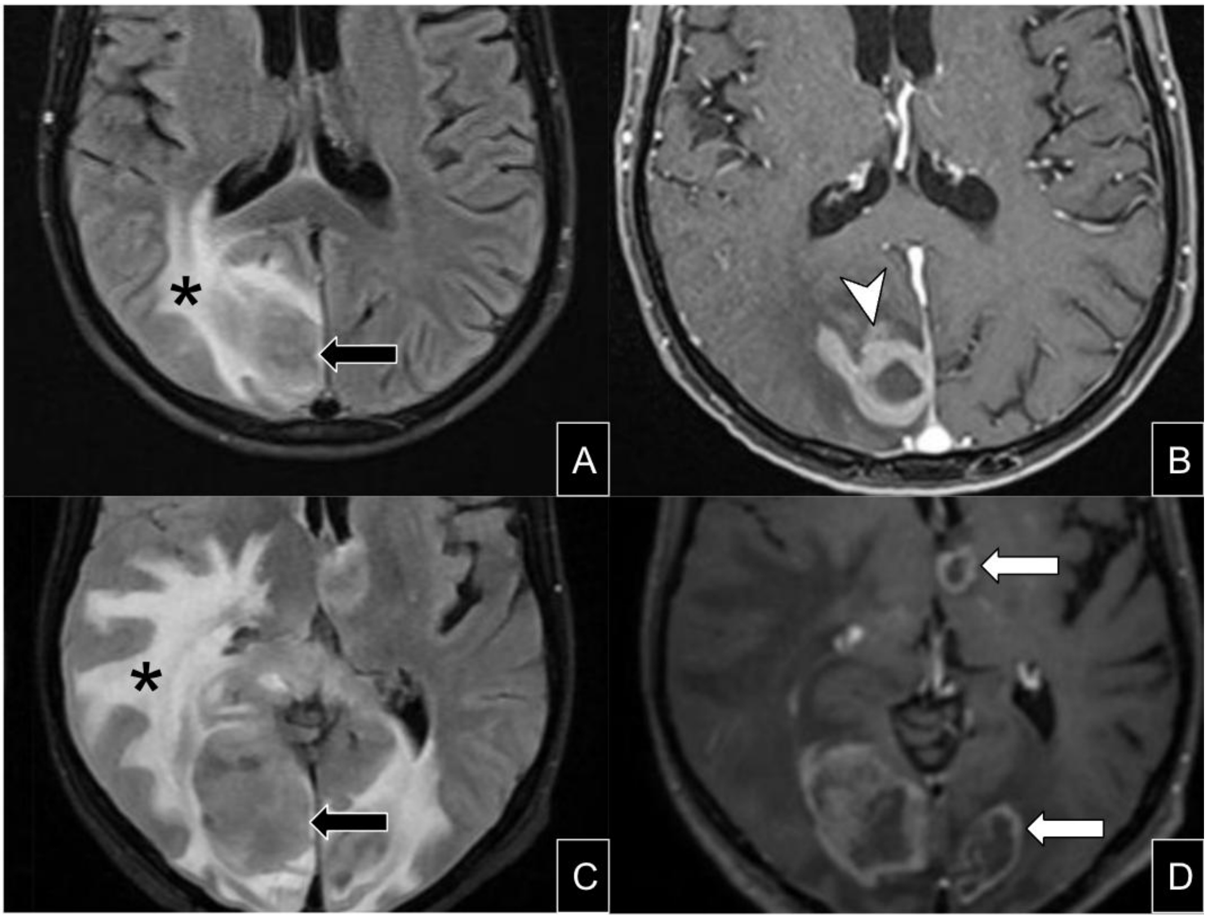

Figure S1. Lymphoma arising in immune deficiency/dysregulation (IDD-lymphoma). Axial FLAIR **(A)** and contrast-enhanced T1-weighted MRI **(B)** display a single subcortical mass lesion (black arrow) in a patient recently diagnosed with HIV, with thick contrast enhancement (arrowhead) and vasogenic edema (asterisk). Follow-up axial FLAIR **(C)** and contrast-enhanced T1-weighted MRI **(D)** after two months with appropriate antiparasitic treatment, demonstrating lesion (black arrows) and edema progression (asterisk), with new thalamic and contralateral lesions (white arrows), related to biopsy-proven IDD-lymphoma.

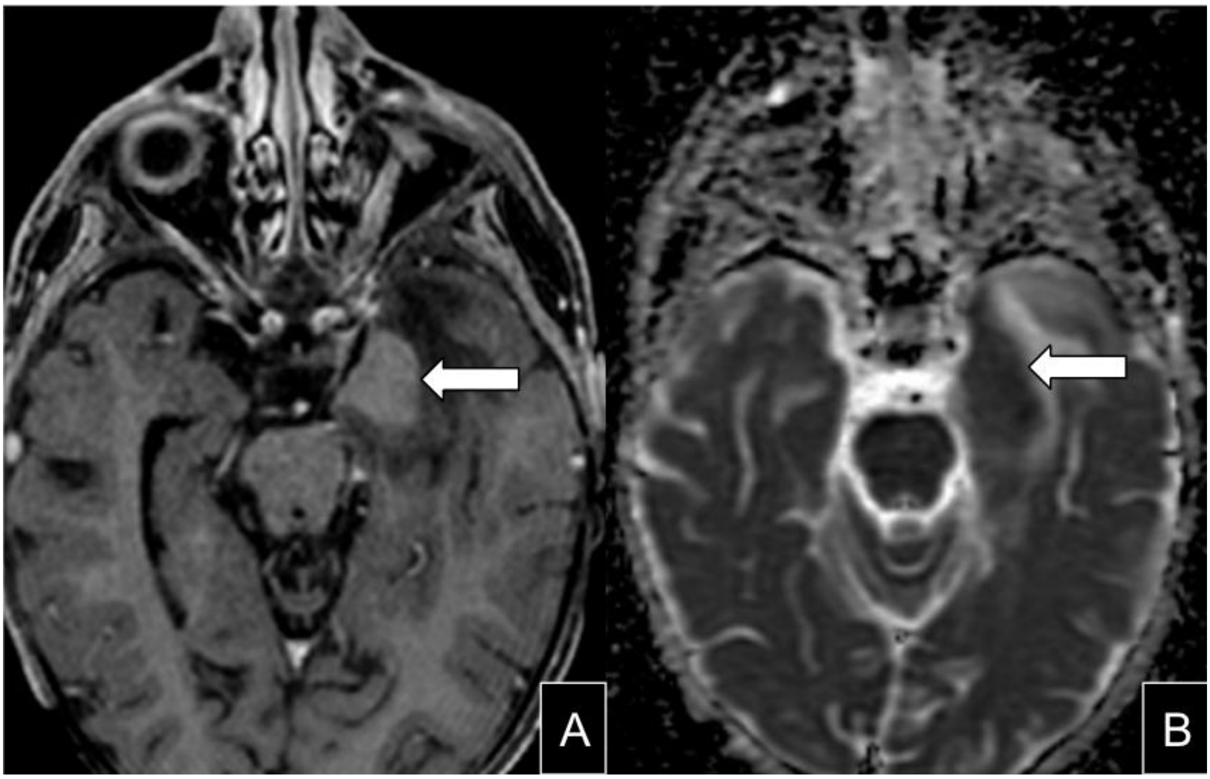

Figure S2. Lymphoma arising in post-bone-marrow-transplant setting. Axial FLAIR **(A)** and ADC **(B)** images in a patient one year after bone marrow transplant presenting with a well-defined and homogenous enhancing lesion affecting the left temporal lobe (black arrow), with low ADC values (white arrow) and vasogenic edema. Biopsy of concurrent lung mass confirmed a post-transplant lymphoproliferative disorder.

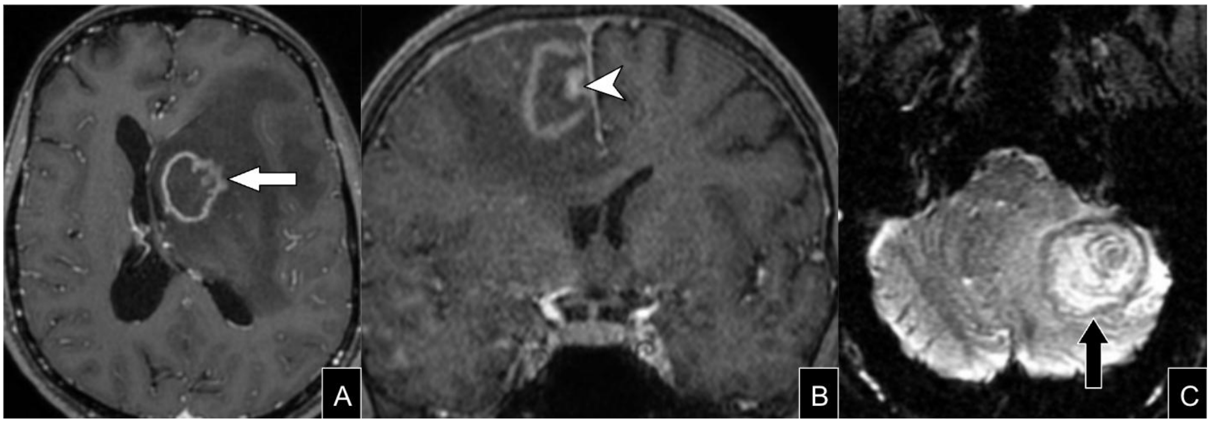

Figure S3. Differential diagnosis of lymphomas arising in immune deficiency/dysregulation (IDD-lymphoma). (A) Axial contrast-enhanced T1-weighted MRI showing a periventricular toxoplasmosis lesion (white arrow) in a patient living with HIV (PLWH), mimicking IDD-lymphoma imaging features. **(B)** Coronal contrast-enhanced T1-weighted MRI shows subcortical neurotoxoplasmosis lesions with eccentric target sign (yellow arrow) in a patient PLWH. **(C)** Susceptibility-weighted imaging of a patient after bone marrow transplant with CNS aspergillosis showing the double rim sign (black arrow).

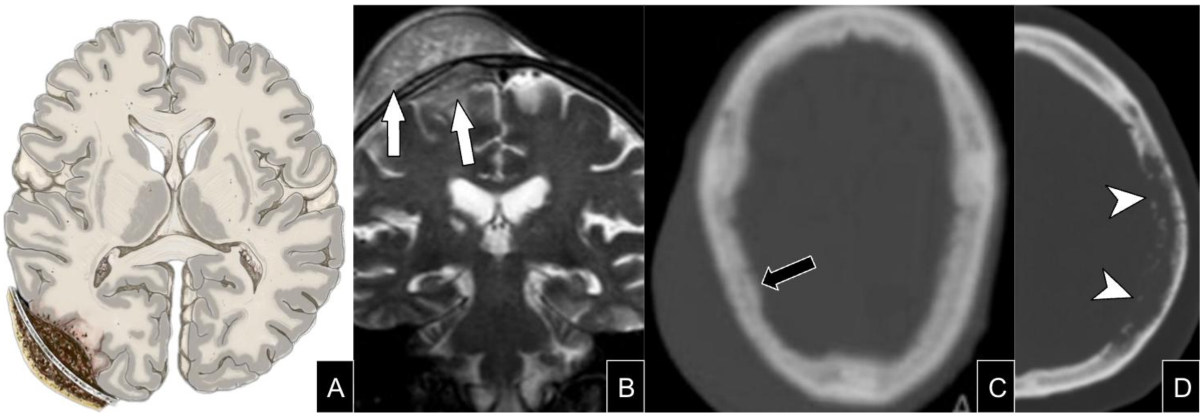

Figure S4. Mucosal-associated lymphoid tissue (MALT) lymphoma of the dura. (A) Schematic representation of MALT lymphoma of the dura mater. **(B)** Coronal MRI of a patient with a solid extra-axial mass in the right frontoparietal high convexity extending extracranially (white arrow). **(C)** Axial CT shows subtle permeative skull changes out of proportion to the lesion's apparent size (black arrow). **(D)** Metastatic breast cancer in a patient with destructive lytic pattern of the skull (arrowheads) for comparison purposes.

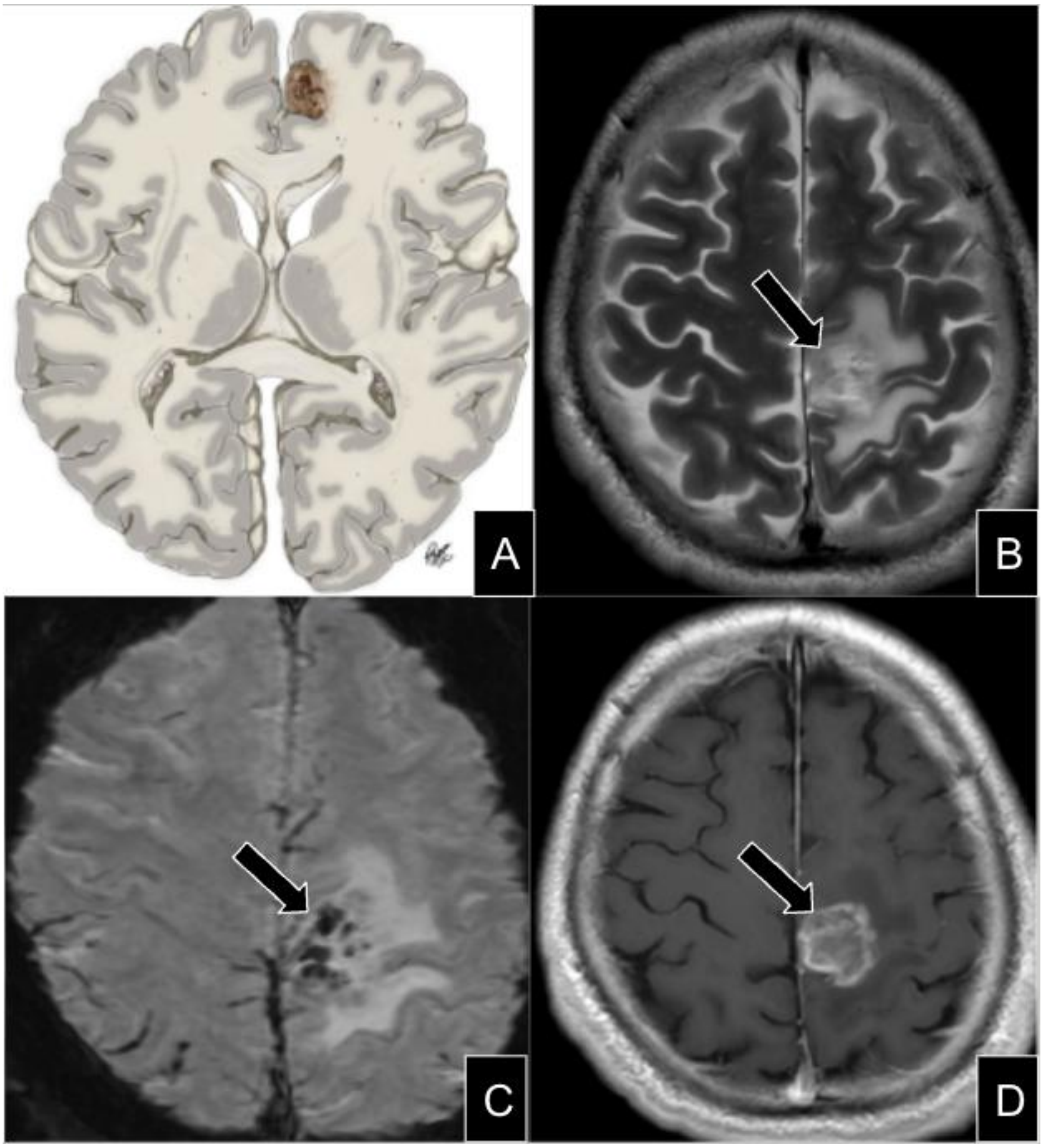

Figure S5. T-cell lymphoma. (A) Schematic representation. **(B)** T2-weighted image in a patient shows focal lesion with high signal intensity and adjacent vasogenic edema (black arrow). **(C)** T2-weighted gradient-echo showing microhemorrhagic foci. **(D)** Post-contrast T1-weighted image demonstrates irregular and heterogeneous enhancement.

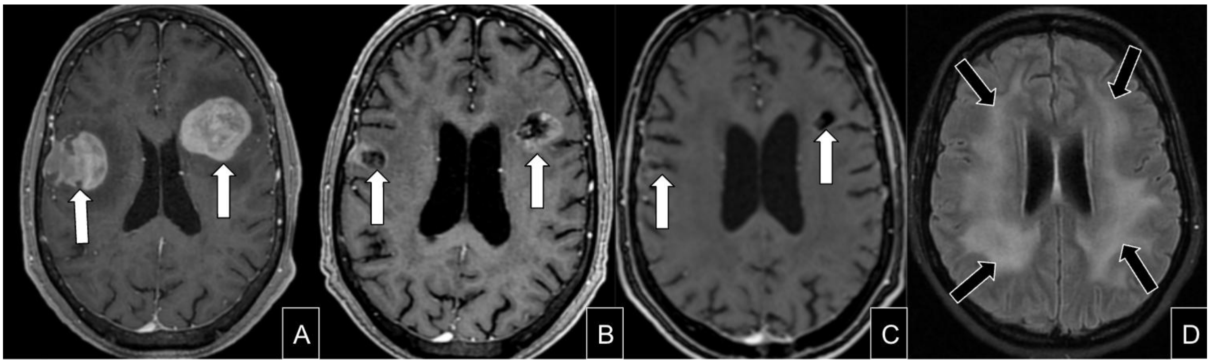

Figure S6. Treatment evolution in large B-cell lymphoma of immune-privileged sites. Serial axial T1-contrast-enhanced MRI **(A-C)** in a patient demonstrating mass lesions (white arrows) centered in frontal lobes. **(A)** Baseline scan shows heterogeneously enhancing lesions with associated mass effect. **(B)** After 9 months of radiochemotherapy, there is marked reduction in lesion volume and contrast enhancement. **(C)** Follow-up study 2 years post-treatment demonstrates complete radiologic remission. **(D)** Axial FLAIR image from a different patient illustrates methotrexate-induced leukoencephalopathy, with confluent periventricular and deep white matter hyperintensities (black arrows).
